# Using dual eye tracking to uncover personal gaze patterns during social interaction

**DOI:** 10.1038/s41598-018-22726-7

**Published:** 2018-03-09

**Authors:** Shane L. Rogers, Craig P. Speelman, Oliver Guidetti, Melissa Longmuir

**Affiliations:** Edith Cowan University, School of Arts and Humanities, Perth, 6027 Australia

## Abstract

We report the personal eye gaze patterns of people engaged in face-to-face getting acquainted conversation. Considerable differences between individuals are underscored by a stability of eye gaze patterns within individuals. Results suggest the existence of an eye-mouth gaze continuum. This continuum includes some people showing a strong preference for eye gaze, some with a strong preference for mouth gaze, and others distributing their gaze between the eyes and mouth to varying extents. Additionally, we found evidence of within-participant consistency not just for *location* preference but also for the *duration* of fixations upon the eye and mouth regions. We also estimate that during a 4-minute getting acquainted conversation mutual face gaze constitutes about 60% of conversation that occurs via typically brief instances of 2.2 seconds. Mutual eye contact ranged from 0–45% of conversation, via very brief instances. This was despite participants subjectively perceiving eye contact occurring for about 70% of conversation. We argue that the subjective perception of eye contact is a product of mutual face gaze instead of actual mutual eye contact. We also outline the fast activity of gaze movements upon various locations both on and off face during a typical face-to-face conversation.

## Introduction

Homo sapiens is the only species of mammal to have evolved eyes with a white sclera (i.e., the large white part of the eye that surrounds the iris and pupil)^1,2^. The unique morphology of the human eye signifies the special role of gaze behaviour for negotiating the subtleties of our social lives. Following gaze direction can facilitate cooperation or deception^3,4^. The ability to signal emotion with one’s eyes further enhances their communicative potential. This was recognized by Baron-Cohen and colleagues when designing their famous ‘reading the mind in the eyes’ test for theory of mind^5^. Specific terms exist within the English vernacular to describe certain types of emotional gaze such as puppy dog eyes (pleading), hungry eyes (lust), and wild eyes (erratic state of mind), just to name a few. An additional important function of gaze is to regulate turn taking behaviour during face-to-face conversation^6^.

Gaze behaviour has received considerable research attention since the 1960s^7^. Over the last few decades developments in eye tracking technology have enabled more nuanced and accurate investigations^8^. However, as pointed out in a review by Pfeiffer, Vogely and Schilbach^9^ the majority of this research has used static eye tracking methods. This involves participants viewing carefully designed stimuli on a computer monitor. While these studies provide high experimental control, generalization of findings to natural conversation is problematic. Risko, Richardson, and Kingstone^10^ have pointed out that in natural contexts gaze behaviour might be different to that observed in studies using static stimuli. They explain that there is a dual function of gaze, as the eyes focus visual attention to gather information for the individual while also potentially communicating information to others. An awareness of the dual function of gaze can influence an individual to behave differently when aware that their eye movements are observable to another person^10–12^.

Recent advances in *mobile* eye tracking technology allow the possibility to study social cognition away from the computer screen. Eye tracking glasses have been developed that allow the tracking of gaze as a participant engages in face-to-face conversation. By utilizing two pairs of glasses, a *dual eye tracking* study can be conducted to obtain a rich data-set. We are only aware of two studies that have examined one-on-one conversation using this procedure in the published literature to date. Broz, Lehmann, Nehaniv, and Dautenhahn^13^ recorded 15 minute getting acquainted conversations between 37 pairs of University students. Each pair of participants wore a pair of Applied Science Laboratory (ASL) eye tracking glasses. The average percentage of the interaction reported to be spent engaged in mutual face gaze was 46%. The findings of Broz *et al*.^13^ were however limited in scope by a lot of missing tracking data. Data loss can occur if a participant is not looking directly through the middle of the lenses (e.g., when making a sideways glance when tilting the head). Ho, Foulsham, and Kingstone^6^ utilized a dual eye tracking paradigm with Dikablis tracking glasses to investigate how timings of gaze on and off the face can regulate turn-taking behaviour during face-to-face conversation. In their study data loss was kept to a minimum by replaying both video recordings synchronously and manually coding behaviour. Evidence for a turn-taking regulatory function of gaze was reported as people tended to begin a speaking turn with averted gaze and ended their speaking turn with a direct gaze toward the partner.

The present study extends upon prior face-to-face dual eye tracking research by utilizing eye tracking glasses (Tobii Pro Glasses 2) and behavioural coding software (Mangold INTERACT). We use this technology to record and present eye gaze patterns during natural conversation at a higher level of detail compared to previous dual tracking research, as we distinguish between different areas both on-face (i.e., forehead, eyes, nose, mouth, and other-face) and off-face (i.e., up, down, off-left, and off-right) similar to prior studies using a static paradigm^14–19^. A study of conversation by Hall *et al*.^20^ took the approach of examining gaze at specific facial features contrasting Fragile X Syndrome participants with controls. An advantage of our approach over that taken by Hall *et al*.^20^ is that the dual tracking nature of our research enables us to report on the typical frequency and duration of both mutual face-gaze and mutual eye-contact during natural conversation. Additionally, the dual tracking approach can provide a more complete picture of gaze behaviour as large glances off-face can be coded and accounted for. Our aims are to: (1) Investigate consistency of gaze patterns within individuals, and explore variation of gaze patterns between individuals (2) Report the prevalence of mutual face gaze and mutual eye contact, and to contrast the perception of mutual eye contact with actual levels of mutual eye contact.

## Consistency of gaze patterns within individuals and variation between individuals

Recent studies investigating eye gaze patterns when viewing images or video of faces on a computer monitor have reported large individual differences^14–19^. Some people gaze predominately at the eyes, some predominately at the mouth, while others distribute their gaze between the eyes and mouth to a varying extent^19^. Within-individual consistency of these personal gaze patterns has been demonstrated to remain stable across testing sessions months apart^18^. Evidence also exists to suggest that within-individual consistency generalizes from computer tasks to real world viewing of faces^17^. In our study we therefore expected to find considerable variation of gaze patterns across participants while at the same time observe consistency of patterns within participants.

## Mutual face gaze and mutual eye contact

In Western culture, it is commonly believed that seeking mutual eye contact with our audience is desirable. This is emphasized most strongly in the human resources and management literature^21^. However, studies of eye contact perception have consistently reported a liberal criterion for judging eye contact that leads people to generally over-estimate how often eye contact occurs^22–26^. A consequence is that the term *mutual eye contact* (two people simultaneously looking specifically at the eyes) is frequently confused with *mutual face gaze* (two people simultaneously looking at the face) in both research literature and common parlance. The present study seeks to add to the research literature by quantifying the amount of mutual face gaze *and* mutual eye contact during face-to-face conversation. Based on the findings of Broz *et al*.^13^ we anticipated that mutual face gaze would comprise approximately 46% of the conversation. No prediction could be made regarding the amount of eye contact as this has not been quantified previously. Based on prior research, however, we could expect that participants would subjectively over-estimate the extent of mutual eye contact^22–25^.

## Results

### Consistency of gaze patterns within individuals and variation between individuals

Of the 49 participants with tracking data, 27 had data for multiple conversations. A visual inspection revealed consistency within participants across these conversations (see online supplement document section-2, and online supplementary data tab ‘all data’). To quantify consistency of gaze patterns when speaking and listening, we performed intra-class correlations (ICCs). ICCs can be used to quantify the extent of clustering within groups that have multiple measurements^27,28^. In the present research, each participant with multiple conversations represents a group of conversation measurements. Due to unbalanced data (i.e., an uneven number of conversations across participants), the ICCs were obtained using the *xtreg* command in the statistical program Stata^29,30^. For the reader interested in the statistics underlying the ICC see pages 80–85 of Rabe-Hesketh and Skrondal^29^. All ICCs are reported in online supplement document section-3. The main finding was a high level of consistency for the proportion of conversation spent gazing at the eyes (ICC = 0.88) and mouth (ICC = 0.80) of one’s partner. This consistency remained when limiting analysis to face gaze only during both speaking (ICC eyes = 0.88; mouth = 0.87) and listening (ICC eyes = 0.84; mouth = 0.84). Consistency was also evident for the mean fixation length on the eyes (ICC = 0.73) and mouth (ICC = 0.69). Lower levels of consistency were generally found for other regions of interest. This is likely due to fixations upon the eyes and mouth being the primary targets of gaze during conversation therefore providing more reliable data compared with other regions.

To explore individual differences between participants we averaged data for participants with multiple conversations to obtain individual scores per participant that were collated with participants with data for a single conversation (see supplementary data tab ‘individual averaged data’). A table of all descriptive statistics is provided in the online supplemental document section-4. Results suggest that during an average getting acquainted conversation half the conversation is spent speaking and the other half listening. The average time per fixation on any location was just under a second for most regions (eyes, mouth, up, down, left right) and half a second or less for the remaining regions (nose, forehead, other-face). In our study gazing away from the face of one’s partner occurred for about 29% of the conversation when speaking, and 10% when listening, a statistically significant difference with a large effect, *t*(48) = 13.79, *p* < 0.001, *d* = 1.82. Prior research has established off-face gaze occurs more frequently during a speaking turn^6,31^. A common explanation is that glancing off-face while speaking frees up cognitive resources for processing self-referential memory during self-disclosure^32^. Despite more frequent off-face gaze when speaking, gaze upon the face dominated the conversation during both speaking and listening turns. During periods of face gazing, on average the eyes and mouth were the primary targets of gaze when both speaking (proportion eyes = 48%; mouth = 29%) and listening (proportion eyes = 43%; mouth = 34%). Comparing speaking with listening, participants generally looked at the eyes more when speaking (*t*(48) = 3.37, *p* < 0.01, *d* = 0.17) and looked at the mouth more when listening (*t*(48) = 3.35, *p* < 0.01, *d* = 0.16). Note however the small effect sizes for these two comparisons. In this paragraph our interpretation of Cohen’s d values is based on Cohen’s^33^ guidelines small (0.2), medium (0.5), and large (0.8).

**Figure 1:**
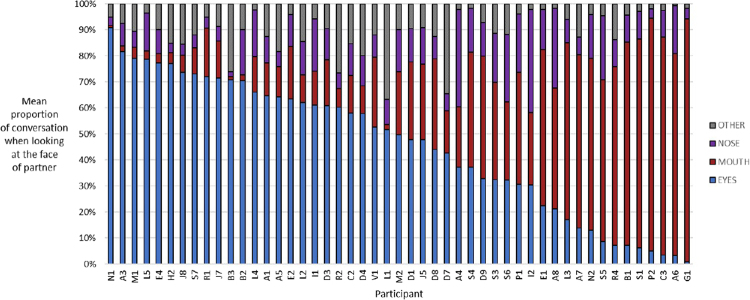
Individual differences in gazing behaviour on the face during conversation. This chart shows each participant’s proportion of gaze spent looking at different facial locations when gazing upon the face of one’s partner during conversation. Participants have been sorted by eye gaze preference to help make the eye-mouth continuum present in the data-set clear. The ‘other’ category represents a merging of the ‘forehead’ and ‘other-face’ categories to simplify the image.

As expected large individual differences were evident. Results suggest the existence of an eye-mouth gaze continuum. This continuum includes some people showing a strong preference for eye gaze, some with a strong preference for mouth gaze, and others distributing their gaze between the eyes and mouth to varying extents. Correlations between proportion of gaze on the eyes (*Spearman r* = 0.93, p < 0.001) and mouth (*Spearman r* = 0.94, p < 0.001) across speaking and listening were very strong. This reveals that a person’s gaze pattern when looking at the face of one’s partner is very consistent within an individual across both speaking and listening. Therefore instead of including two separate charts we averaged across speaking and listening to illustrate individual differences across participants regarding the extent of eye and mouth gazing when looking on-face during conversation, see Fig. 1. No association was found between gender with proportion of gaze towards the eyes or mouth. Age was found to negatively correlate with proportion of gaze towards the eyes (*Spearman r* = −0.35, *p* < 0.05) and positively correlate with proportion of gaze towards the mouth (*Spearman r* = 0.40, *p* < 0.05). Looking over scatterplots revealed these associations were influenced by a small sub-group of four participants over-55 years old with a low level of gaze towards the eyes (with a corresponding prominent level of mouth gaze). These scatterplots are provided in the online supplement document section-4. When excluding these participants, associations diminished (*Spearman r* = −0.17 and 0.23, respectively) and were no longer statistically significant. Results suggest that mouth gazing might be more common in older adults perhaps due to hearing troubles that lead them to engage in more lip-reading. However, age did not predict preference for eye or mouth gaze in participants under 55 years old.

### Actual and perceived mutual face gaze and eye contact

Our methods enabled a quantification of both mutual face gaze (i.e., both parties simultaneously looking at the face of their partner) and eye contact (i.e., both parties simultaneously looking at the eyes of their partner). Mutual face gaze averaged 63% (*SD* = 16, *min* = 27, *max* = 88) of conversation for episodes averaging 2.2 seconds in length (*SD* = 1, *min* = 0.7, *max* = 4.7). Mutual eye contact averaged only 12% (*SD* = 12, *min* = 0, *max* = 46) of conversation for episodes averaging 0.36 seconds in length (*SD* = 0.2, *min* = 0.1, *max* = 0.9). These averages must be interpreted with caution for two reasons. First, the range for both types of mutual gaze are large, particularly for eye contact. The extent of mutual gaze is dependent upon the gaze behaviour of both parties involved in the conversation. If two people come together who gaze predominately at the eyes then eye contact will likely be high (*max* = 46% of conversation), however if a single member (or both) of the pair are more inclined to gaze at the mouth then mutual eye contact will likely be very low (*min* = 0% of conversation). Secondly, due to the small number of conversations (*n* = 51, see supplementary data tab ‘Mutual Gaze’) and unbalanced nature of our data (i.e., some participants contributed to more conversations than others) more research with a larger sample size is required to provide more generalizable findings.

After each conversation participants were asked to rate their perceived extent of mutual eye contact. We averaged responses across conversations for each participant, and then averaged these scores across the participants (see supplementary data tab ‘individual averaged data’). The extent of perceived mutual eye contact was consistently “often” or “very often” (*Mean* = 5.5, *SD* = 0.7, *min* = 3.3, *max* = 6.0). We recognise that measuring perceived eye contact on this scale is difficult to match up to our percentage figures for actual behaviour. We therefore collected additional data from 212 (*Mean* age = 24, *SD* = 10, *min* = 17, *max* = 72, 73% female, see supplementary data tab ‘Est. eye contact (1)’) first year university students who participated in a round-robin session of four 4-minute getting acquainted conversations with their peers during a lecture time slot. After each conversation students rated perceived mutual eye contact on the same scale used in the lab study. Responses were averaged for each student across their four conversations to obtain a single score per student. Results from this new group of participants (*Mean* = 5.4, *SD* = 0.6, *min* = 2.8, *max* = 6.0) did not significantly differ from the eye tracking sample (*Independent t* = 1.11, *p* = 0.27). This established that the perceived levels of eye contact were similar across the in-lecture activity and our lab study. We then replicated the in-lecture activity with an additional group of 171 1^st^ year students (*Mean* age = 24, *SD* = 9, *min* = 17, *max* = 60, see supplementary data tab ‘Est. eye contact (2)’) and instead of the never - very often scale we used a scale of 0–100% with options in 10% increments. As expected, this sample also typically reported perceiving a lot of mutual eye contact during their conversations (*Mean* = 72%, *SD* = 16, *min* = 8, *max* = 100). Considering our estimates of actual mutual eye contact (ranging from 0–46%), the present study provides evidence to suggest that during face-to-face conversation in general people greatly over-estimate the extent of *mutual eye contact*. Their estimates more closely align with (although still slightly over-estimate) *mutual face gaze*.

## Discussion

This study represents an examination of eye gaze behaviour during face-to-face conversation achieved by utilizing eye tracking technology (Tobii glasses 2) and behavioural coding software (Mangold INTERACT). We aimed to examine consistency of eye movement patterns and the prevalence and subjective perception of mutual face gaze and eye contact.

### Consistency of eye gaze patterns

Recent studies have shown consistency in eye scanning patterns when looking at pictures or video of faces^14–19^. Our study extends upon this work by finding that consistent eye gaze patterns also occur during face-to-face conversation. The same individual differences observed by Kanan *et al*.^19^ emerged in our study. That is, participants differed on a continuum based on their proportion of fixations upon the eyes or mouth. When looking at the face some people directed their gaze mostly towards the eyes, some predominately at the mouth, and some distribute their gaze upon the eyes and mouth usually in a rapid upwards-downwards pattern. We also found evidence of within-participant consistency for the *duration* of fixations upon the eye and mouth regions. These gaze patterns remained consistent within individuals during episodes of speaking and listening. The interested reader can observe the within-participant consistency and between-participant variability of gaze patterns in the visualizations provided in the online supplement document section-2.

What consequences individual differences in gaze patterns might have for attention, memory, and social functioning during conversation poses a large area for future inquiry. We have demonstrated that consistent gaze patterns emerge across multiple conversations with different partners in a single context (i.e., getting acquainted conversation). How different situational factors such as mood, conversational goals/topics, relationship status between individuals, and number of people conversing influences deviations away from one’s default pattern is an intriguing area for future research^34–37^. Dispositional influences are also worth further investigation. For example, based on prior research there is likely to be differences in what constitutes the most typical default patterns across cultures^38,39^. As another example, the largest investigation of individual difference thus far using a static paradigm has reported gender differences with females more likely to distribute gaze between the eyes and mouth to a greater extent compared with males^15^. Future research investigating gaze patterns during conversation will require a larger sample size to what we have reported in this study to adequately explore the influence of both dispositional and situational influences.

### Mutual face gaze and mutual eye contact

A decisive advantage of a dual tracking paradigm is the possible examination of mutual face gaze^13^ and mutual eye contact. Broz *et al*.^13^ previously reported approximately 46% of 15-minute conversations to comprise mutual face gaze (i.e., both parties simultaneously looking at the face of their partner). In 4-minute getting acquainted conversations we found that approximately 60% of the conversation comprised mutual face gaze. The difference between the prevalence of mutual face gaze in our study compared with Broz *et al*. could be due to a difference in conversation time lengths (i.e., 15-min conversations in Broz *et al*. versus our 4-min conversations), or because of differences in coding procedures and equipment. Additionally, we found that episodes of mutual face gaze occurred on average for approximately 2.2 seconds. A recent study showing images of directly gazing faces to participants reported the typical preferred length of mutual face gaze to be approximately 3.3 seconds^40^. Our results therefore reveal that the typical period of mutual gaze during actual conversation is perhaps a full second shorter than that found to be preferred when using more static methods.

The present study is the first to quantify mutual eye contact (i.e., both partners simultaneously looking at each other’s eyes). Due to the broad variation among individual eye gazing behaviour towards the eyes and mouth regions there was unsurprisingly a corresponding very large variation in the prevalence of mutual eye contact across conversations. When an individual who engages in a prominent level of mouth gazing (with associated low eye gazing) is part of the conversation eye contact will be very limited. We found that eye contact ranged from 0–45% across different conversations that varied on participant eye-mouth gazing preferences. The overall mean was 10% of conversation comprising mutual eye contact, with an average length of 0.36 seconds. Due to our relatively small sample size (i.e., 51 conversations) and unbalanced data (i.e., some participants contributed to multiple conversations) our results must be interpreted with caution. More research is needed to provide a more robust estimate regarding the prevalence of mutual eye contact, and how this might be influenced by other factors. For example, personal attraction might increase seeking of mutual eye contact^41,42^.

Our results suggest that mutual face gaze typically comprises a high proportion of face-to-face conversation and mutual eye contact is fleeting. Yet our study also revealed the subjective perception of eye contact to be high at around 70%. Our results therefore suggest an over-estimation of eye contact during face-to-face conversation. This is consistent with research that has used more static paradigms to demonstrate that when an individual is uncertain about where their partner is gazing there is a bias to perceive gaze directed towards one’s eyes^22–26^. We argue that participants are generally ‘blind’ to subtle eye movements and instead ‘see’ high levels of mutual eye contact. We argue that this perceived high level of mutual eye contact is a consequence of high levels of mutual face gaze, instead of eye contact per se. Our results therefore suggest that sensitivity regarding specific eye movements upon facial locations is very limited. Perhaps the cognitive demands of conversation dedicated to speech comprehension and production limit cognitive resources available for close attention to subtle deviations in the eyes of one’s partner. A limitation of our study is that our specific post-conversation questions asked only about the eyes (rather than other locations) that may have artificially increased participant estimates of eye contact. Additionally, some participants simply might not make a conceptual distinction between mutual face gaze and mutual eye contact when they appraise mutual eye contact. More research is clearly required to help confirm our assertions regarding a lack of sensitivity to subtle eye movements during natural conversation.

### Active gaze during face-to-face conversation

In addition to providing evidence for a lack of sensitivity to subtle eye movements, another finding of the present research is to reveal what might be referred to as the ‘active eye’ during face-to-face conversation. Here we are inspired by research investigating the ‘quiet eye’ during sports performance. The quiet eye refers to a calm, steady and extended gaze during specific motor actions, such as hitting a ball or shooting at a target^43^. In conversation, our results indicate that the eyes of both partners are generally very active. Any location is typically fixated on for less than a second before moving to another location for an equally brief fixation, before moving on, and so on. We must also acknowledge that there are exceptions with prolonged gaze instances occurring that are worthy of future research in themselves. Regardless, the generally quick eye movements during conversation appears to be outside of the conscious awareness of both parties. We suggest the fast activity of the eyes represents non-conscious scanning of facial features during interaction. Facial features can communicate subtle indicators of the emotion and meaning behind the words being spoken. Such as the slight upturn of the corner of the mouth, a narrowing of the eyes, or a twitch of an eyebrow^44^. Do those with more active eyes pick up on these subtle cues, either consciously or non-consciously? This is an intriguing avenue for future research.

### The impact of wearing eye tracking glasses on eye gaze behavior

A common question we have received when giving talks about our gaze tracking research is whether wearing the eye tracking glasses might have an impact on gaze behaviour of participants. For example, do participants become anxious knowing their gaze is being tracked? Based on our experience running the data collection sessions the participants outwardly seemed very comfortable and enjoyed the conversations with their university peers. If anyone was concerned about having their gaze tracked, arguably those concerns would be difficult to maintain when faced with the cognitive demands associated with conversing with a previously unacquainted partner. Does talking with another person wearing the glasses impact where participants look or how they perceive the other person? It has been reported that wearing glasses might attract the gaze of an observer and influence the wearer to be perceived as more intelligent^45^ and honest^46^, yet not necessarily more attractive^47,48^. However, research on the effects of wearing glasses has relied on judgments of static photographs. As face-to-face eye tracking research grows in popularity, exploring the potential impact of wearing eye tracking glasses during face-to-face social encounters would assist to clarify if wearing glasses (when previously unaccustomed to them) may or may not impact the gaze behaviour, comfort, and impression formation of wearers in dual tracking research.

There are several implications of this emerging technique of dual eye tracking for increasing the understanding of socio-cognitive processes during conversation. Apart from the obvious benefit of further understanding everyday communication, there are multiple other fields of research that can benefit. For example, further understanding the role of eye gaze in different contexts such as tasks requiring joint attention^49,50^, education^51^, social anxiety^52^, other psychological disorders^20,53,54^, and the creation of socially capable artificially intelligent systems^55^.

## Method

### Participants

A total of 76 undergraduate students participated in the research, which involved having a varying number of one-on-one face-to-face conversations with other participants. There were 176 conversations overall, and as each conversation could produce two sets of gaze data (i.e., a set of gaze data for each of the two participants having a conversation), there were hypothetically 352 sets of gaze data obtainable. This study only reports data from conversations where an individual was *accurately tracked* with the Tobii Glasses (we explain how this was determined in the procedure section). We acquired 107 sets of accurately tracked gaze data, collected from 49 participants (*mean age* = 32 years, *SD* = 14, *min* = 17 & *max* = 67). Some reasons why our final set of data was substantially smaller than what could have been acquired all related to the fact this was our first experience using the Tobii Glasses: (1) We did not screen out participants who needed corrective lenses, as we were recruiting participants for another research project that was running in parallel to the study reported here. There are corrective lenses than can be purchased for use with the Tobii Glasses however we did not possess that option during our data collection. (2) For some participants we did not compensate for a slight downward tilt in the Tobii Glasses camera that meant in some instances the visual recording only captured half the head of the person they were conversing with, so these instances had to be discarded. (3) We were conservative with our criteria for accepting the tracking as accurate. Prior to commencement, the research was approved by the Edith Cowan University Human Research Ethics Committee. All participants provided informed consent. This research was performed in accordance with the National Statement on Ethical Conduct in Human Research as outlined by the Australian Government National Health and Medical Research Council (NHMRC).

### Equipment

To simultaneously record eye gaze patterns of two participants we used two pairs of Tobii Pro Glasses 2 (50 Hz model: http://www.tobiipro.com/product-listing/tobii-pro-glasses-2/). The eye tracking glasses contain a high definition camera and microphone embedded in the middle of the glasses for recording the audiovisual scene in front of the wearer (resolution of 1920 × 1080 pixels, at 25 frames per second). The audiovisual recording is stored on a recording unit that connects to the glasses. The wearer’s eye gaze behaviour is tracked and recorded via four sensors with a 50 Hz sampling rate. The manufacturer has reported a spatial accuracy of 0.63 degrees at a distance of 1.5 metres^56^. This manufactory testing is conducted under ideal conditions so this is likely to be an over-estimation of the spatial accuracy quality in our study^57^. In our study participants are seated across from each other at 1 metre distance with much of the conversation people staring straight ahead. Compared to other mobile tracking studies where participants are moving around an environment with large head movements^17^, our study arguably represents a context relatively close to the testing benchmark context. The glasses require dedicated Tobii software to operate and analysis software to export the recordings as .mp4 files. We also used specialized behavioural coding software to manually code gaze behaviour that allows synchronous playback and manual coding of two audiovisual files – Mangold INTERACT (https://www.mangold-international.com/en/software/interact).

### Procedure

Participants were recruited to visit the ECU Cognition Research Group lab and engage in a short round robin of 4-minute getting acquainted conversations. Participants were not instructed to speak about any specific topics, instead they were left to their own devices to ensure the conversation be as natural as possible. The ideal was 4 people attending a session with everyone having 3 conversations. This however was difficult to achieve due to participants not showing up, or sometimes having to leave the session early. Also, a handful of participants returned for a second or third session. We analyze eye tracking data from the 49 participants for whom we successfully and accuractely recorded gaze patterns. These participants engaged in a varying number of conversations that ranged from a single conversation up to seven conversations.

During conversation, participants sat directly across from one another 1 metre apart. To provide an assessment of the accuracy of gaze tracking, we utilised a make-shift test board by taping a piece of paper over a clapper-board with a 1, 2, and 3 drawn at the top-left, middle, and bottom-right of the board, respectively. The board was held up in front of the face of the conversational partner, and the participant was instructed to gaze at the 1, 2, and 3 as the numbers were spoken aloud by the experimenter (each digit spoken with a pause of about 2 seconds afterwards). This was then repeated for the participant who had just had the board held in front of their face (i.e., The board was held up in front of the face of their conversational partner and the numbers were counted). For an example of accurate, and inaccurate tracking, please see online supplement document section-1. After the accuracy check, the clapper-board was used to signal the start of the conversation by performing the clap in-between the two participants. After each conversation, participants rated their perceptions of mutual eye contact via the question ‘There was mutual eye contact between myself and my partner’. This question was rated on a 6-point scale: Never, Very rarely, Rarely, Sometimes, Often, Very often.

#### Coding eye gaze fixations

To examine participant eye gaze patterns, we exported the Tobii glasses footage as .mp4 files using Tobii analysis software. These audiovisual files contain an eye tracking overlay in the form of a red circle that represents a person’s attentional focus at any point in time. The Tobii software allows for the tracking circle to be exported at varying sizes. We decided on a size of 15 pixels as the ideal size for our purposes. This size was decided as ideal because it provided balance between distinguishing different coding regions of interest, while also maintaining comfort for the coder when watching the playback. Smaller sizes would require the coder to squint when watching the playback that would cause discomfort over the course of coding hours. The Tobii analysis software provides an option for a fixation filter to be applied to the tracking data and overlay. The Tobii I-VT fixation filter classifies gaze into fixations where the tracking is stable upon a small window based on parameters of space, velocity, and time. In our research we used the default parameters set by the Tobii Analyzer software prior to exporting videos as .mp4 files. Specifically, the default settings used were: Gap fill-in (interpolation) -> Max gap length = 75 ms; Noise reduction -> Moving median, window size (samples) = 3; Velocity calculator -> Window length = 20 ms; I-VT classifier -> Threshold (°/s) = 30; Merge adjacent fixations -> Max time between fixations = 75 ms, Max angle between fixations = 0.5; Discard short fixations -> Minimum fixations duration = 60 ms. For a full explanation of parameters see Tobii Technology online White Paper^58^. The sensitivity of the filter means it does not remove blinks.

Pairs of videos were opened using the Mangold INTERACT software. The use of a clapper-board enabled the recordings to be played back in-sync after adjusting start times appropriately. A participant’s eye gaze behaviour was manually coded using INTERACT according to the location being fixated upon at any point in time for several on-face locations (forehead, eyes, nose, mouth, & other-face) and off-face locations (up, down, off-left, & off-right). ‘Other-face’ refers to the cheeks and jaw areas of the face, essentially any spot not covered by the other locations. ‘Eyes’ included both eyes and in-between the eyes. When a participant blinked, this was included in the preceding code. For example, if a participant was gazing at an eye of their partner this would be coded as ‘eyes’, and if the participant blinked and shifted to ‘off-left’, that blink would have been coded as part of the preceding ‘eyes’ code. Our methods also allowed us to quantify the extent and timing of mutual faze gaze (i.e., both partners simultaneously looking at each other’s face) and eye contact (i.e., both partners simultaneously looking at each other’s eyes). In general, videos were played back frame-by-frame, but for some participants with less variable gaze movements the video could be played back faster. Coding of gaze behavior took approximately 35 minutes (ranged from about 20–60 minutes) per person within a conversation. Coding the 107 sets of gaze data therefore took roughly 62 hours.

**Figure 2:**
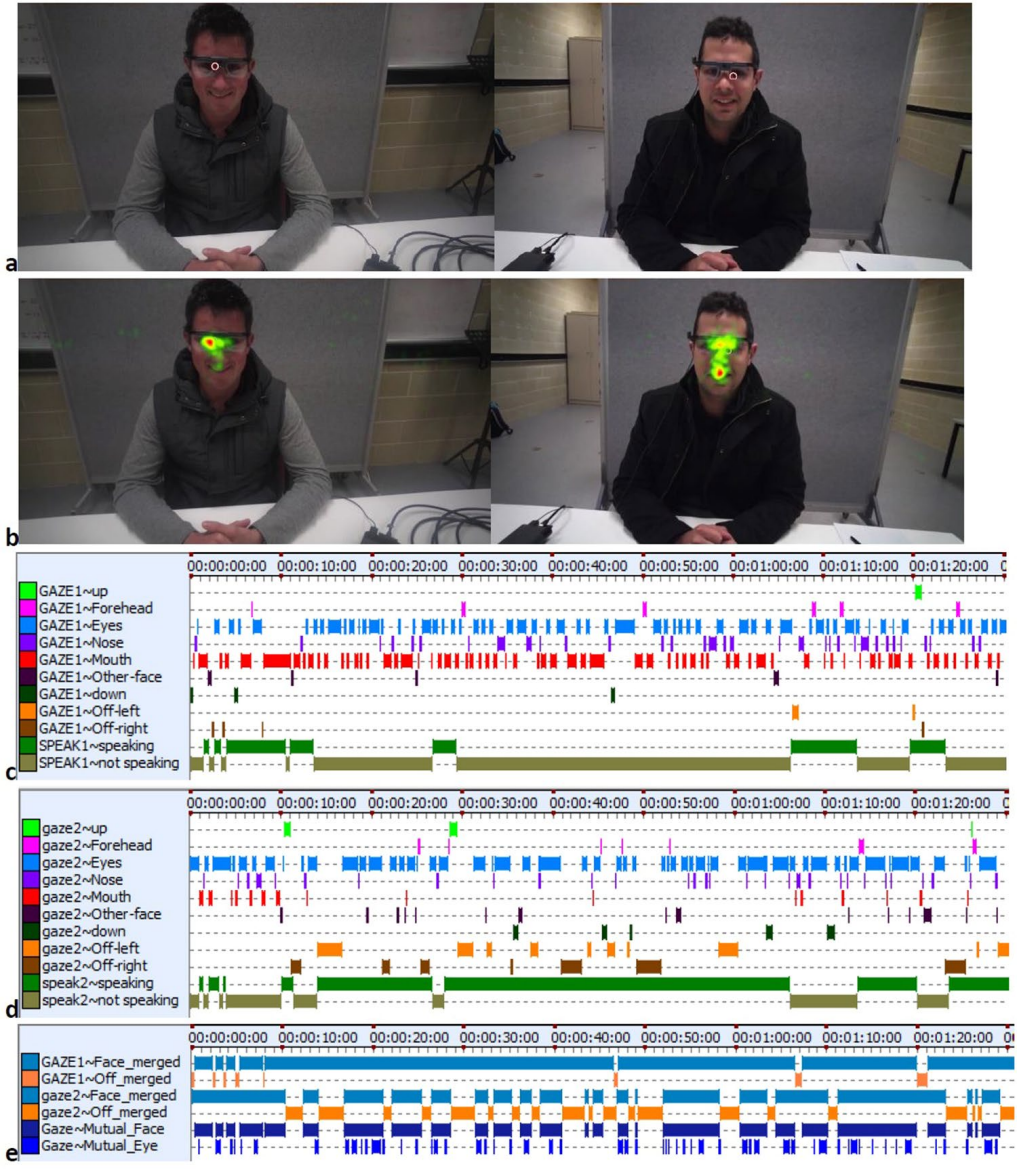
A visualization of eye gaze patterns. (**a**) Snapshots of participants D1 (left) and I1 (right) taken from the footage captured by the Tobii glasses. The small red circle represents attentional focus as captured by the Tobii glasses. Note that this snapshot of conversation represents an instance of mutual eye contact between the two participants. The Participant images shown are used with permission granted by the participants. (**b**) Gaze heatmap overlay of fixation duration for participant I1 gazing at D1 (left), and D1 gazing at I1 (right), for the entire 4-minute conversation. Warmer colour (red/orange/yellow) indicates more gazing time, and cooler colour (green) indicates less gazing time. Using Tobii Pro Analyzer software the head movement of participants was accounted for in the heatmap images. (**c**) Gaze pattern, and speaking/listening time, for participant D1 (looking at I1) for the first minute and a half of conversation. (**d**) Gaze pattern, and speaking/listening time, for participant I1 (looking at D1) for the first minute and a half of conversation. (**e**) On and Off face gaze pattern, for the first minute and a half of conversation, for participant D1 (labelled ‘GAZE’) looking at I1, and for participant I1 (labelled ‘gaze2’) looking at D1. The two blue patterns at the bottom of the image represent periods of mutual face gaze, and mutual eye contact, respectively. The full 4-minute representation of gaze patterns are provided in the online supplemental document section-2.

For each conversation, each participant was coded separately, however the ability to watch simultaneous video playback from both participants using INTERACT was useful for coding purposes. This is because playing the two video recordings synchronously side by side allows identification of off-face gaze behaviour that is difficult to determine otherwise. This is because off-face glances can at times fall outside the tracking capability of the glasses. When this occurred, the dual video footage allowed the coder to determine where the large off gaze was directed (e.g., off-left). Large sidelong glances were clearly visible when looking at the video footage being played back from the point of view of the conversational partner. Using the visualization capability of INTERACT we can provide a detailed picture of an individual’s eye gaze pattern, see Fig. 2. All eye gaze pattern visualizations are provided in online supplement document section-2.

We also coded all interactions for speaking and listening turns. Back-channel verbal responses such as “Mmm” and “Yeah” were not treated as a speaking turn and were instead coded as part of a listening turn. Therefore, speaking turns specifically represent verbal acts of questioning or self-disclosure. Coding of speaking/listening turns took approximately 15 minutes (ranged from about 10–40 minutes) per person. Coding the 107 sets of speaking/listening data therefore took roughly 27 hours. Inter-rater reliability was assessed by a separate independent person coding the full 4-minute conversation eye gaze patterns of the participants D1 and I1 that are shown in Fig. 2. Cohen’s Kappa was calculated using the INTERACT software, and was found to be satisfactory for both participant D1 (Kappa = 0.68) and I1 (Kappa = 0.70) for eye fixation coding. This was also the case for D1 (Kappa = 0.78) and I1 (Kappa = 0.92) for speaking/listening coding. In this article we adhere to guidelines for interpreting Cohen’s Kappa and intra-class correlation: Poor (less than 0.39), fair (0.40–0.59), good (0.60–0.74), and excellent (0.75–1)^59^.

### Data availability

Please see supplement material associated with this article.

## Electronic supplementary material

Online Supplement Document

Supplementary Dataset 1
