# Supplementary material for: Using dual eye tracking to uncover personal gaze patterns during social interaction
Source: Sci Rep. 2018 Mar 9;8:4271. doi: 10.1038/s41598-018-22726-7 (PMC5844880; doi:10.1038/s41598-018-22726-7)

Online Supplement file.

For Scientific Reports article:
Using dual eye tracking to uncover personal gaze patterns during social interaction

Authors:
Rogers, S. L., Speelman, C. P., Guidetti, O., and Longmuir, M.

Affiliation (all authors):
Edith Cowan University
School of Arts and Humanities
Perth, Australia

Corresponding author:
Shane L. Rogers
shane.rogers@ecu.edu.au

PLEASE NOTE THIS DOCUMENT CONTAINS FOUR SECTIONS.

SECTION 1. Assessing eye tracking calibration accuracy, page 2.

SECTION 2. Eye tracking and speaking/listing coding visualisations for each participant, for each of their conversations, pages 3-28.

SECTION 3. Intra-class correlations, pages 29-34.

SECTION 4. Descriptive statistics, pages 35-36.

SECTION 1. Assessing eye tracking calibration accuracy.

Example video stills when assessing the accuracy of gaze tracking from an ‘acceptable’ participant (A, B, C), and from an ‘unacceptable’ participant (D, E, F). Prior to starting the conversation, the board was held up in front of the conversational partner’s face and the participant was instructed to fixate on the 1, 2, and 3 as the numbers were read aloud (a pause of about 2 seconds was held before stating the next number in the sequence).

A)

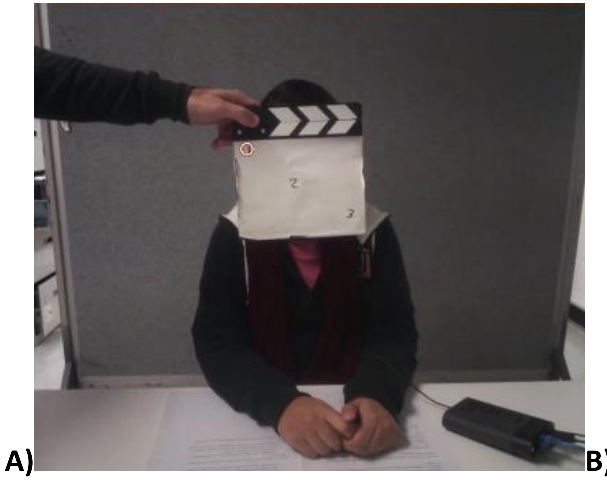

B)

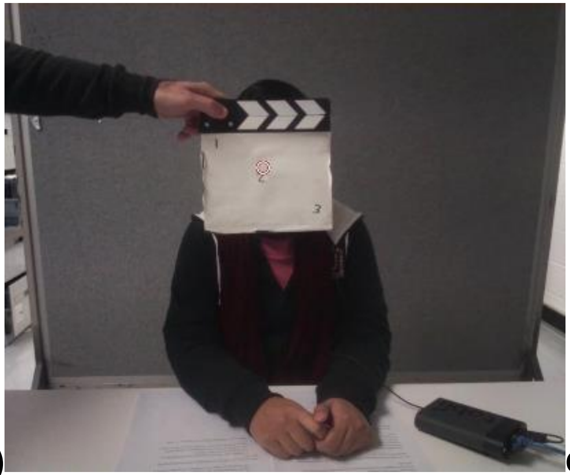

C)

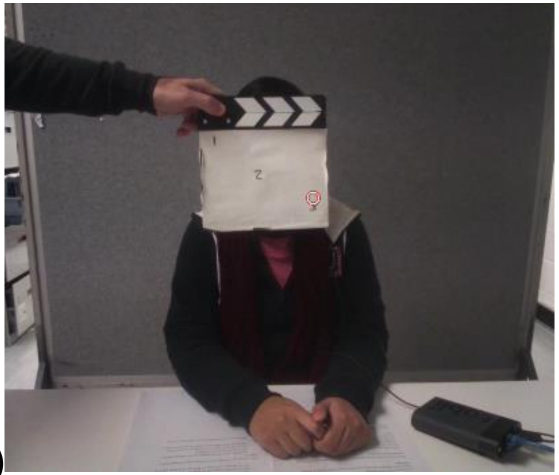

D)

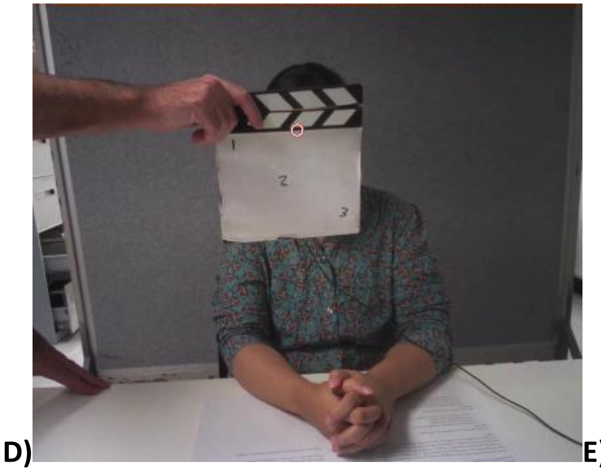

E)

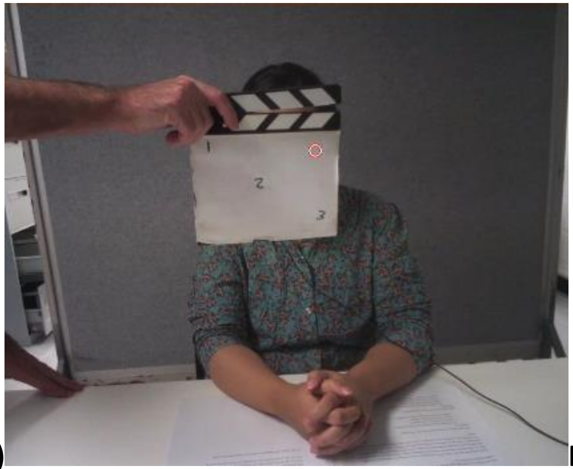

F)

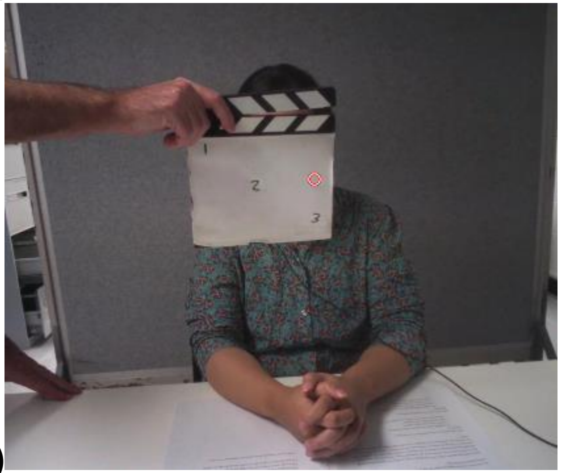

SECTION 2. Eye tracking and speaking/listening coding visualisations for each participant, for each of their conversations.

*This supplemental document for article ‘***Using dual eye tracking to uncover personal gaze patterns during social interaction***’ provides the eye gaze pattern visualisations and speaking/listening turns for each participant with tracking data. Under each participant listing (in bold) are all visualisations obtained for that participant. For example, the first participant shown below ‘A1’ has their visualisations shown when interacting with participant B3 (in session 10), with B1 (in session 19), and G1 (in session 19). Both the coding and visualisations on the subsequent pages were obtained using behavioural coding software Mangold INTERACT:* https://www.mangold-international.com/en/products/software/behavior-research-with-mangold-interact

Participant A1 (F, 22)

S10 With B3 (F, 22)

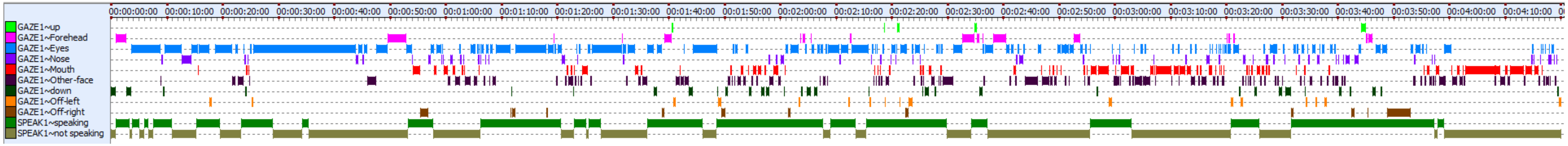

S19 with B1 (M, 18)

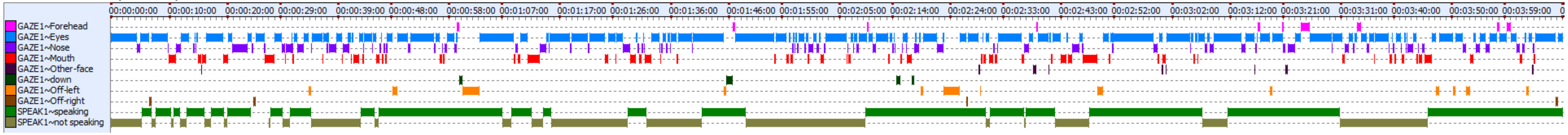

S19 with G1 (M, 60)

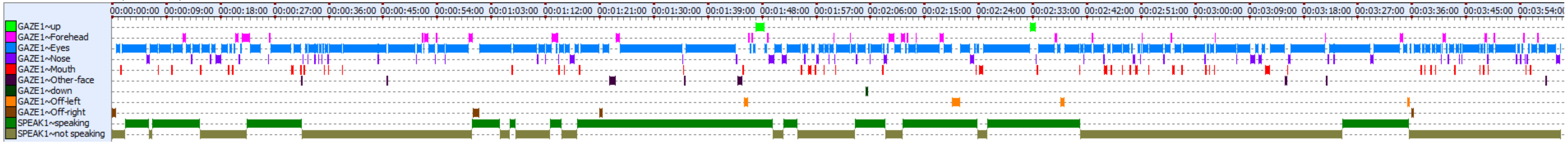

Participant A3 (F, 27)

S13 With G1 (M, 60)

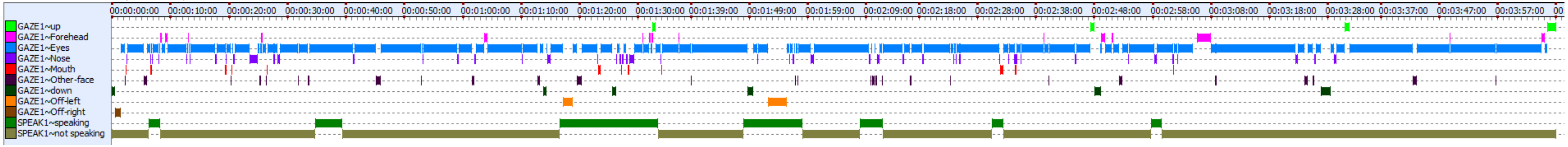

Participant A4 (F, 50)

S17 With N1 (M, 22)

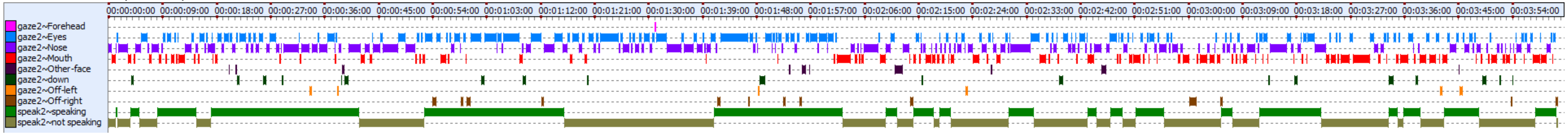

Participant A5 (F, 18)

S21 with D1 (M, 32)

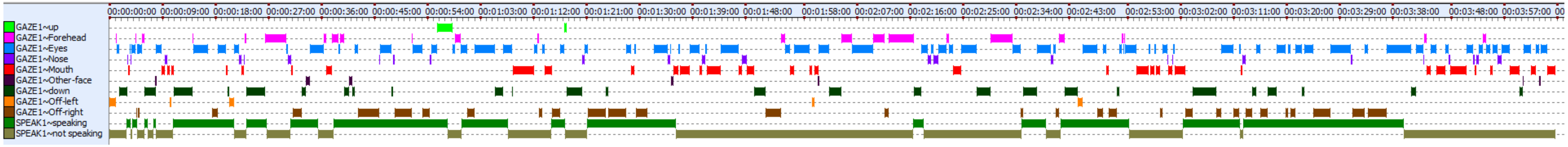

S21 with I1 (M, 28)

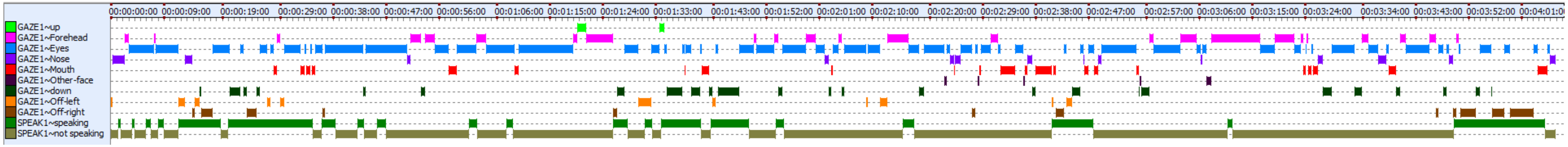

S21 with S5 (F, 39)

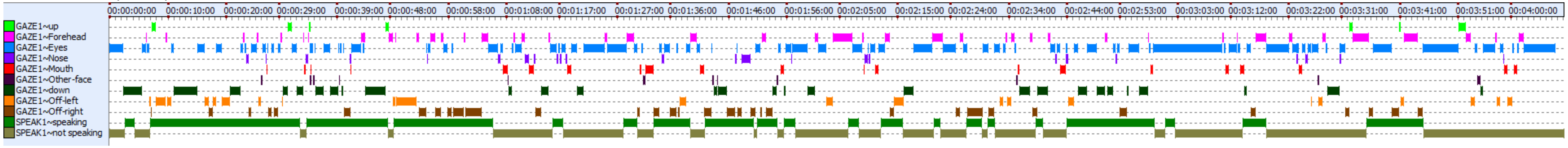

Participant A6 (M, 23)

S22 with R1 (F, 18)

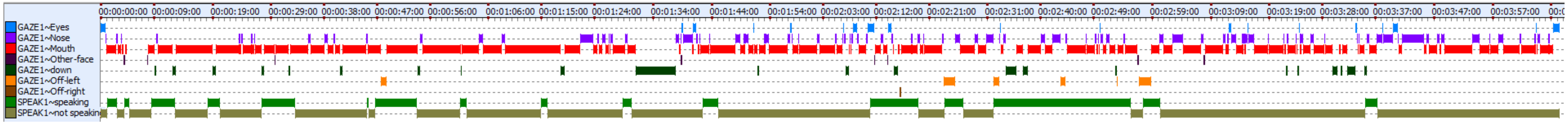

Participant A7 (F, 27)

S23 with C2 (F, 20)

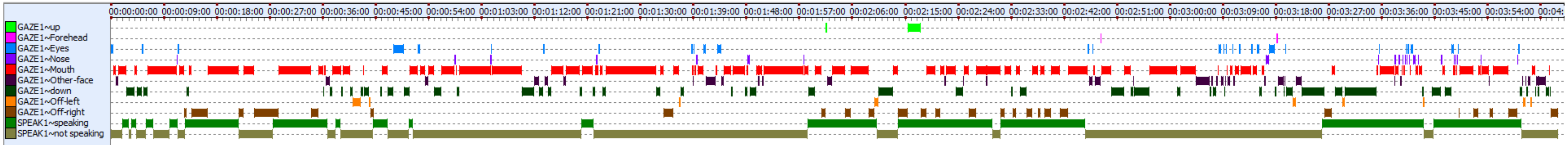

S23 with D4 (M, 45)

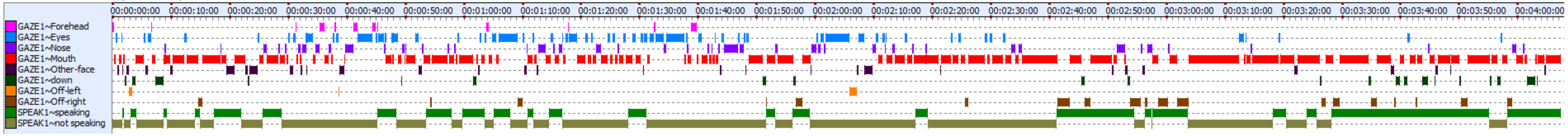

S23 with J5 (F, 50)

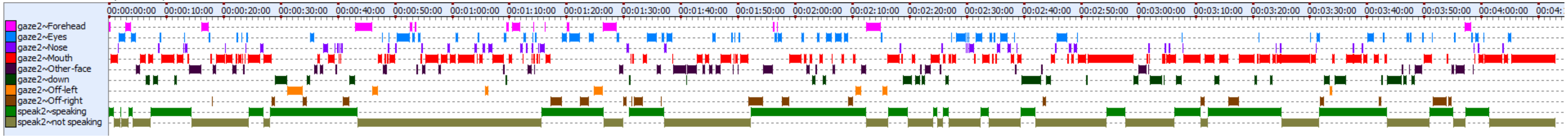

Participant A8 (F, 40)

S26 with L4 (M, 25)

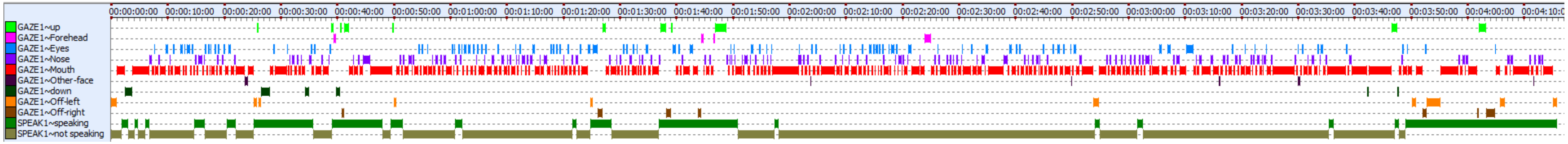

S26 with J8 (F, 17)

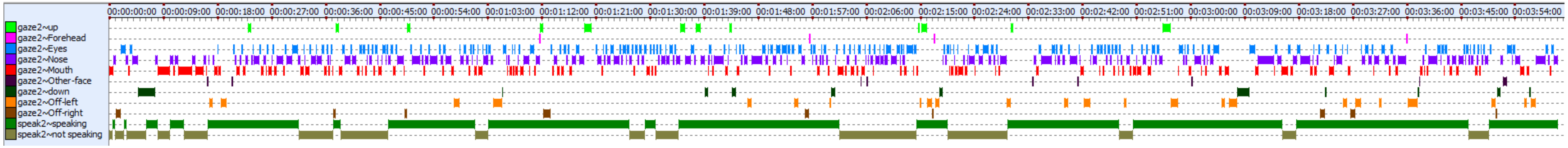

Participant B1 (M, 18)

S6 With L1 (M, 18)

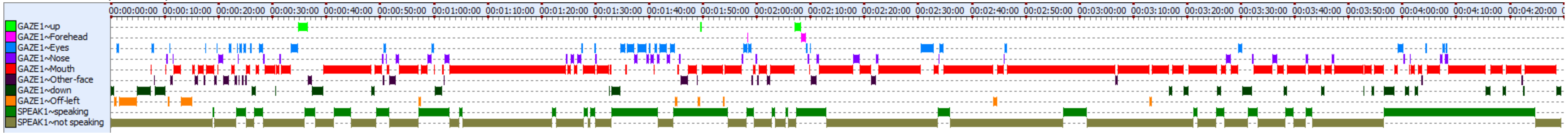

S18 with R1 (F, 18)

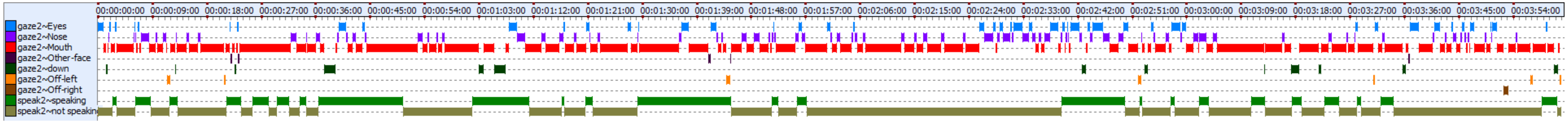

S19 with A1 (F, 22)

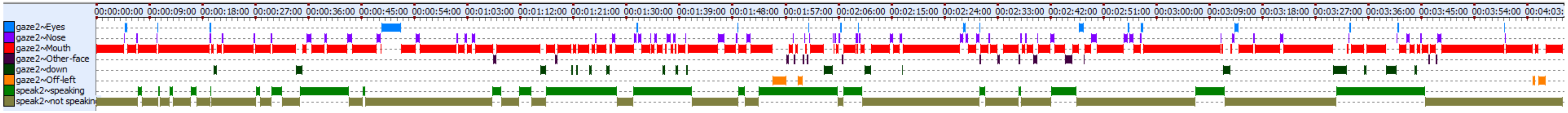

S19 with G1 (M, 60)

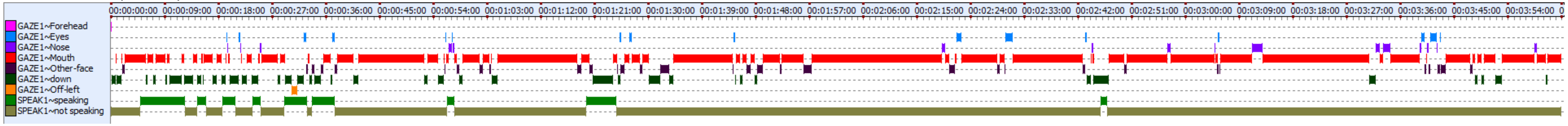

S19 with S4 (M, 29)

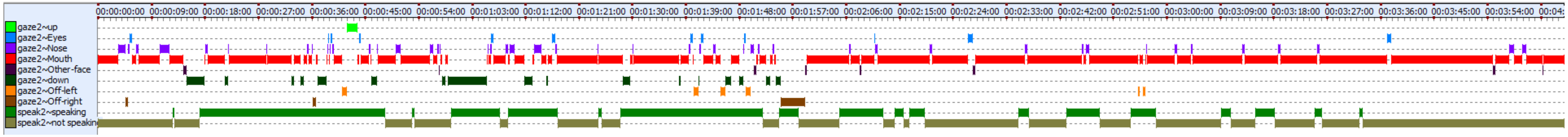

Participant B2 (F, 19)

S9 With P2 (M, 67)

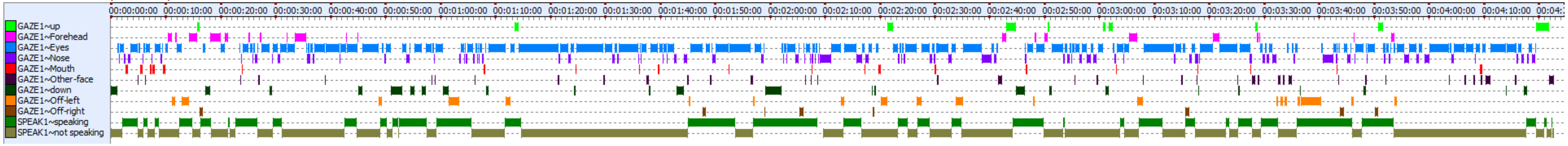

Participant B3 (F, 22)

S10 With A1 (F, 22)

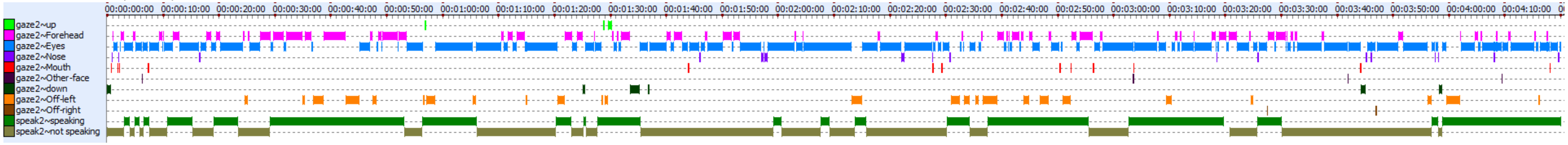

Participant C2 (F, 20)

S23 with A7 (F, 27)

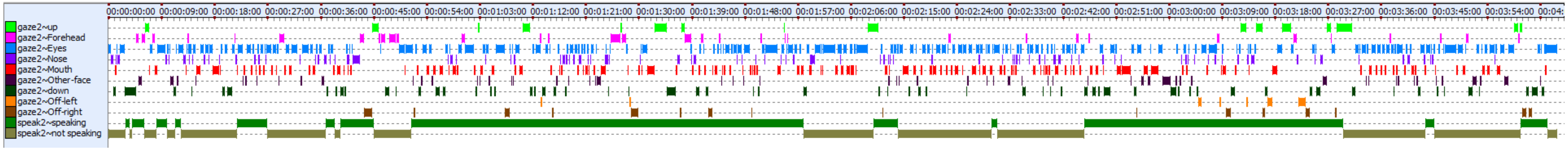

S23 with D4 (M, 45)

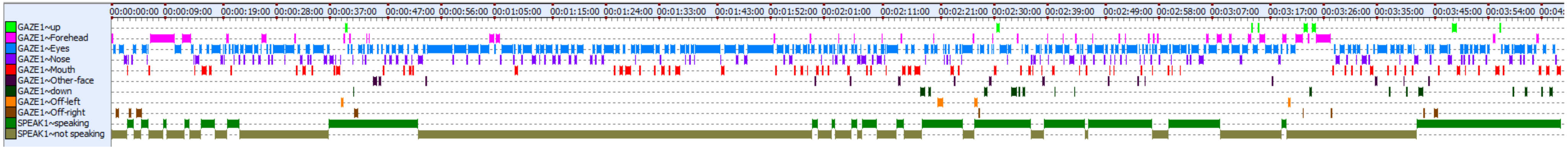

S23 with J5 (F, 50)

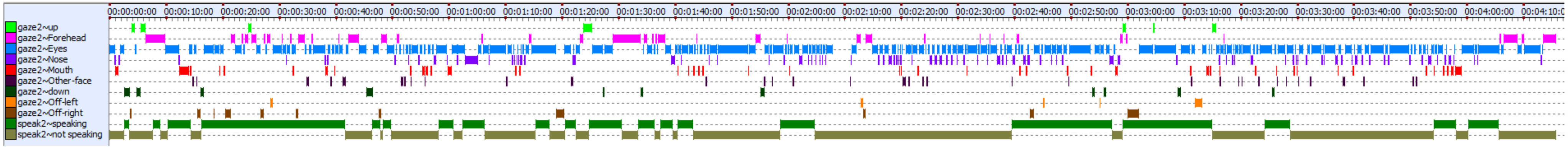

Participant C3 (M, 59)

S30 with L5 (M, 36)

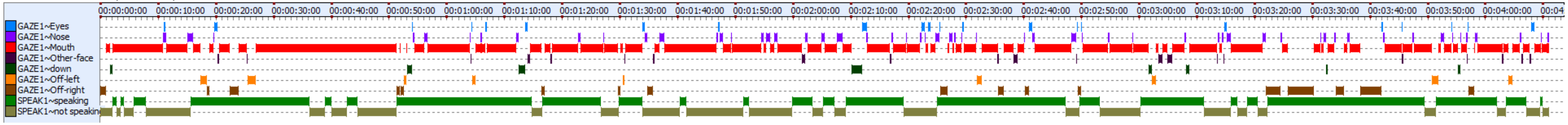

Participant D1 (M, 32)

S1 With I1 (M, 28)

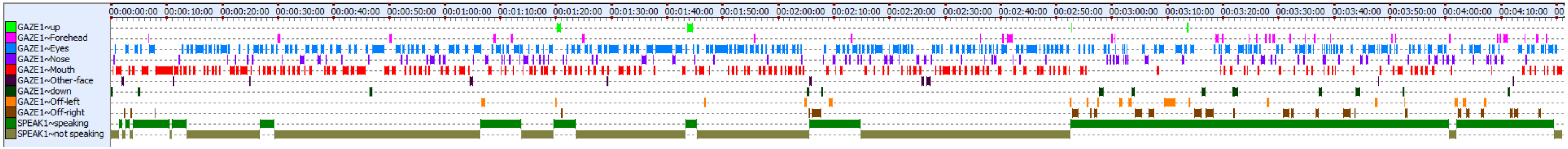

S21 with A5 (F, 18)

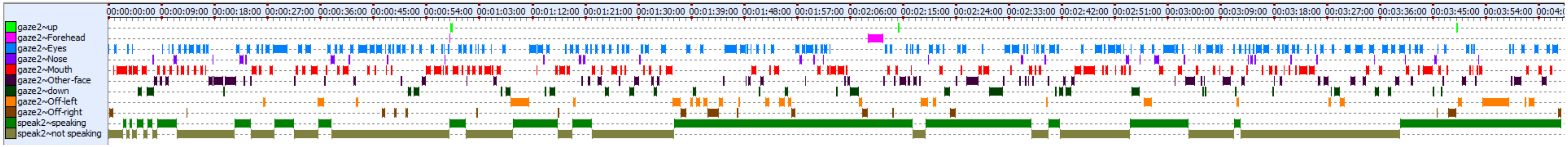

S21 with S5 (F, 39)

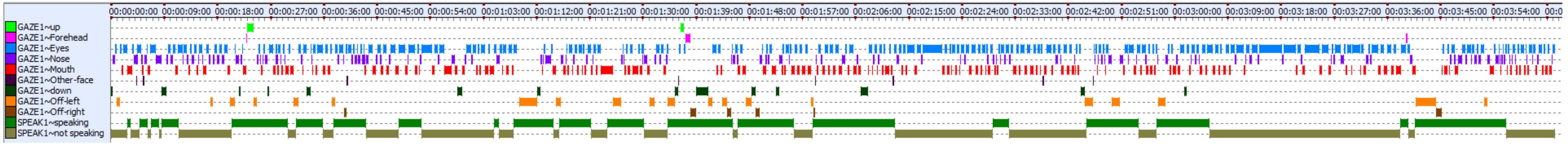

Participant D3 (F, 45)

S8 With P1 (F, 52)

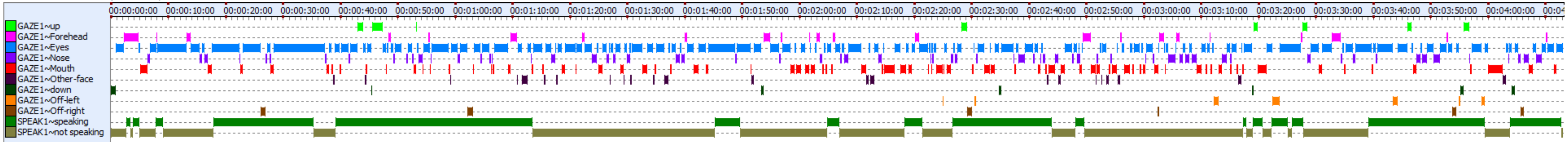

Participant D4 (M, 45)

S23 with A7 (F, 27)

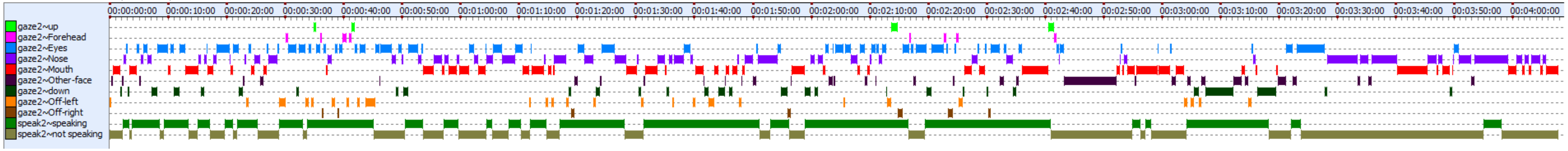

S23 with C2 (F, 20)

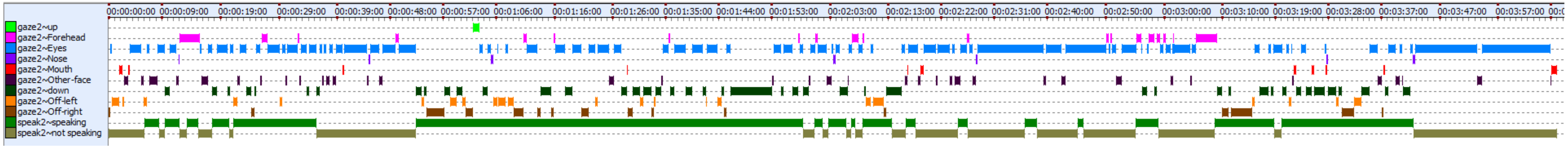

S23 with J5 (F, 50)

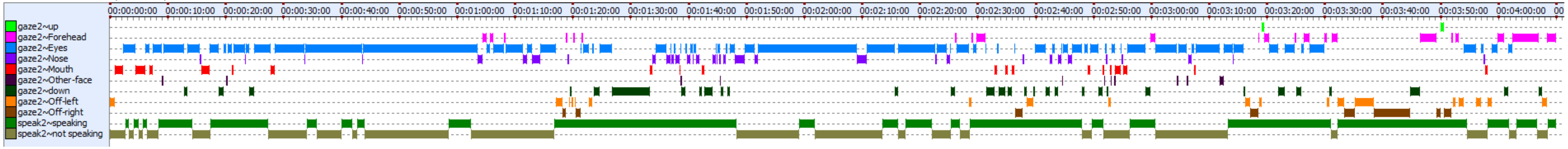

Participant D7 (M, 19)

S27 with H2 (M, 20)

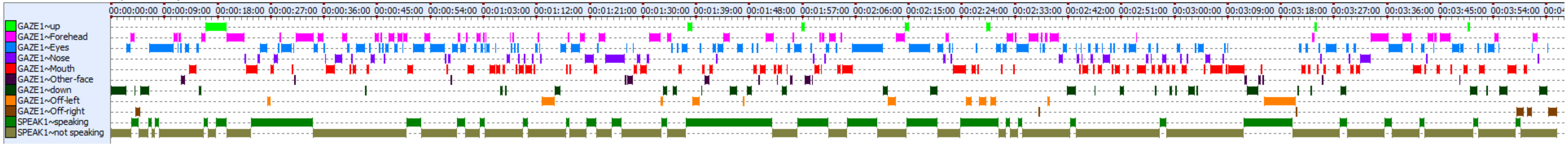

S27 with J8 (F, 17)

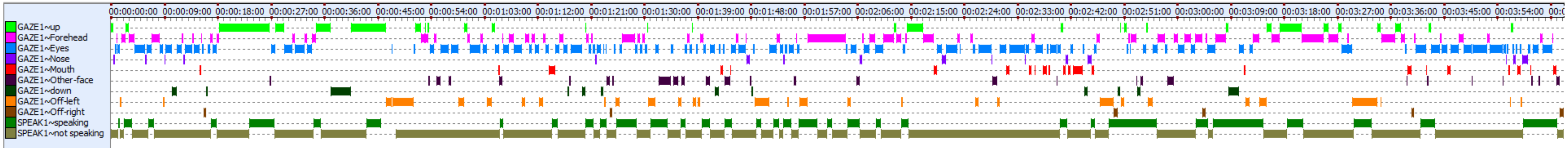

S27 with N2 (F, 35)

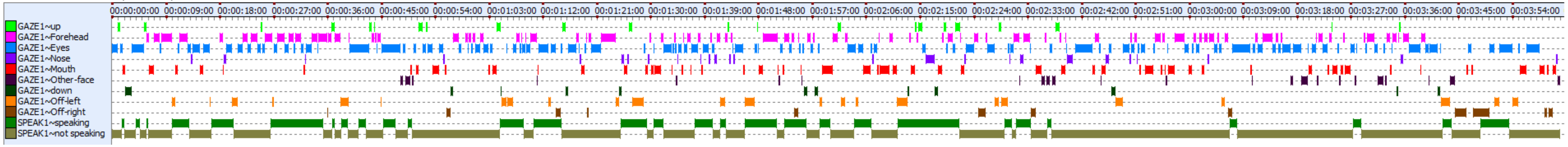

Participant D8 (F, 22)

S29 with R4 (M, 38)

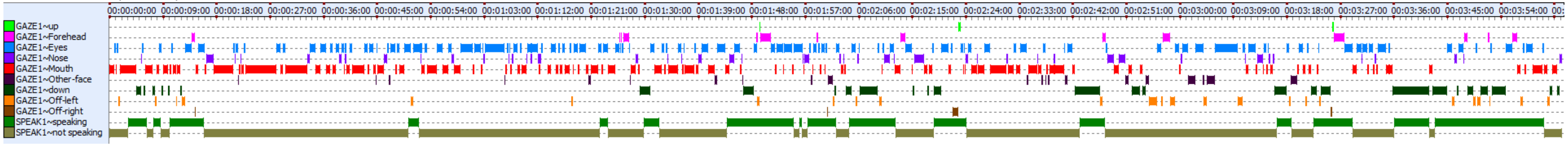

Participant D9 (F, 20)

S30 with S7 (M, 48)

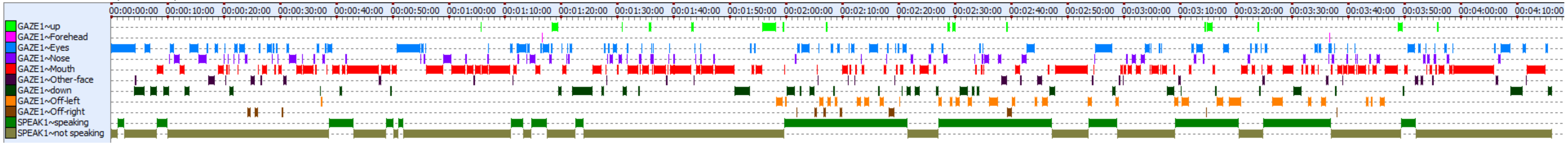

Participant E1 (F, 20)

S24 with J6 (F, 21)

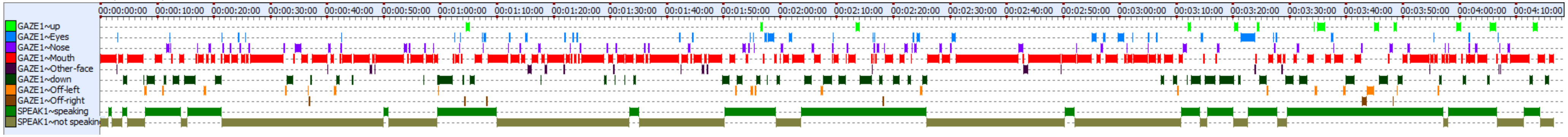

S24 with K1 (F, 18)

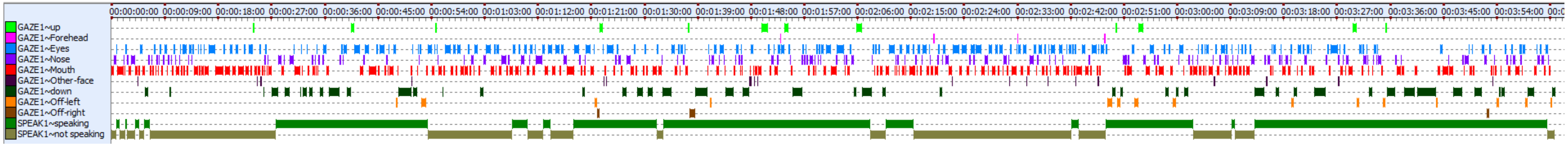

Participant E2 (F, 40)

S26 with L4 (M, 25)

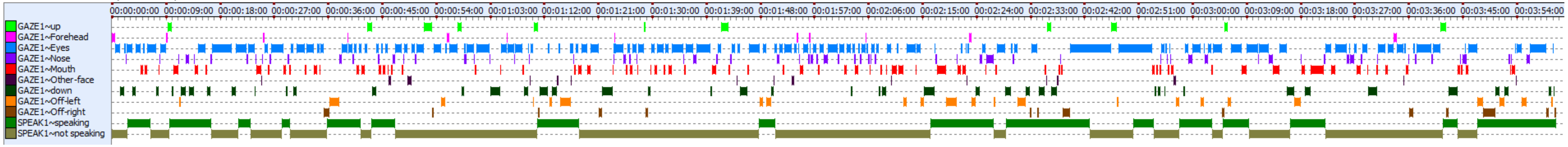

Participant E4 (F, 20)

S28 with J9 (M, 20)

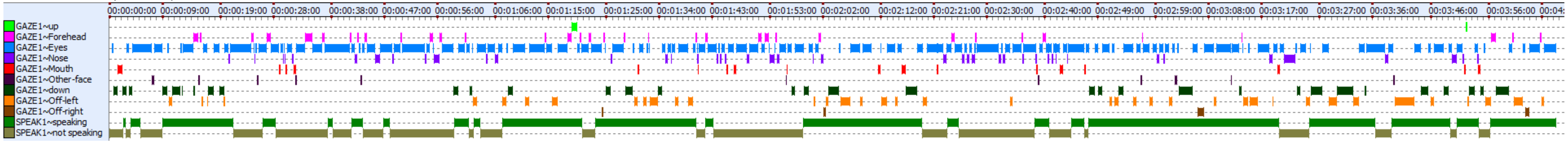

Participant G1 (M, 60)

S13 With A3 (F, 27)

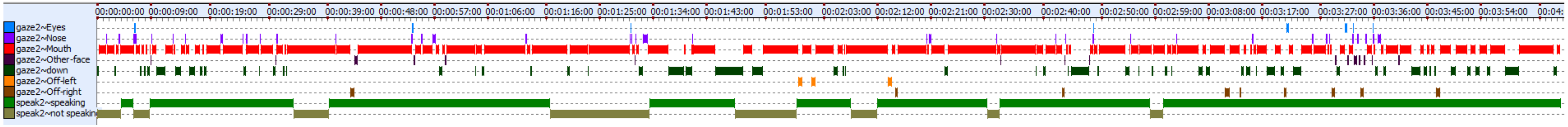

S18 with S4 (M, 29)

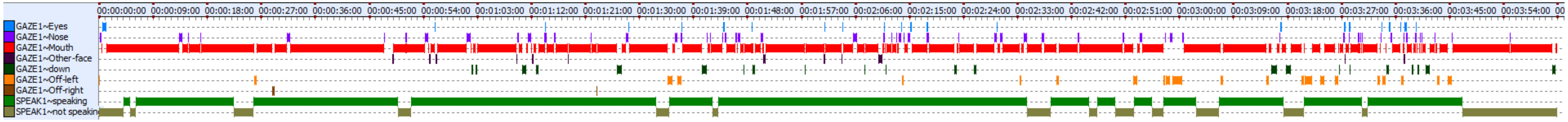

S19 with A1 (F, 22)

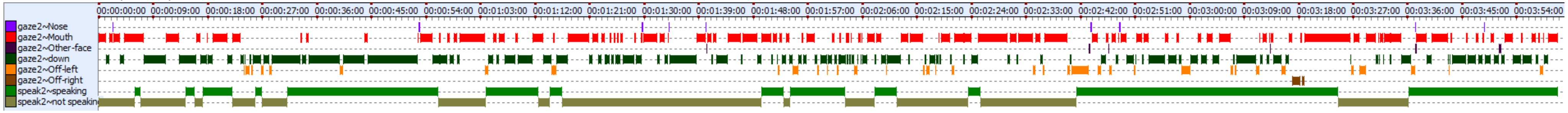

S19 with B1 (M, 18)

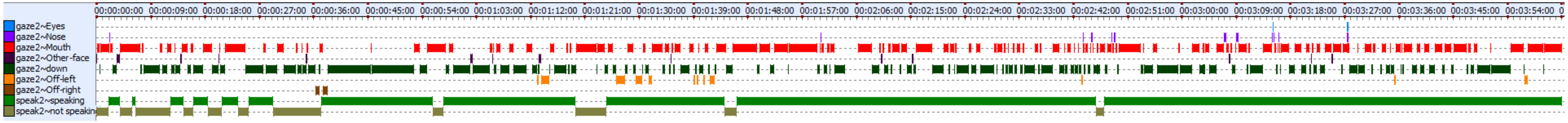

S19 with R1 (F, 18)

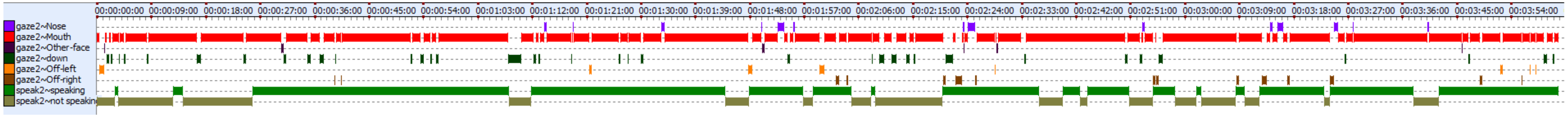

S20 with L2 (F, 20)

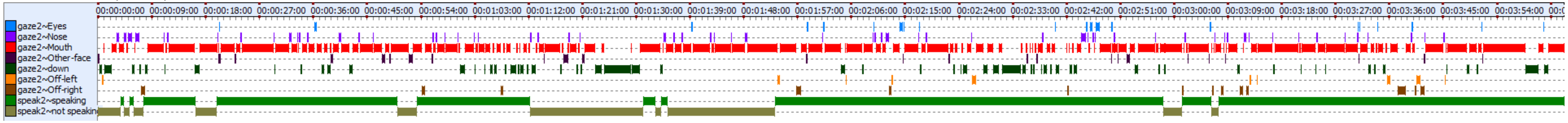

S20 with M1 (M, 42)

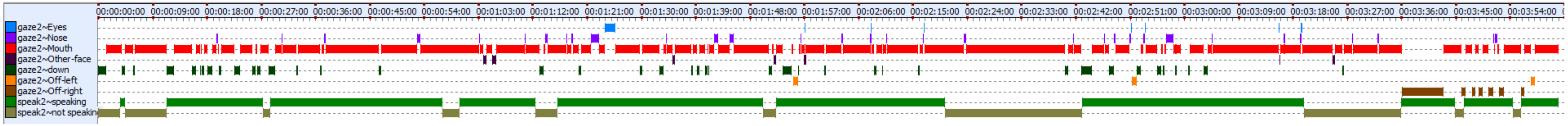

Participant H2 (M, 20)

S27 with D7 (M, 19)

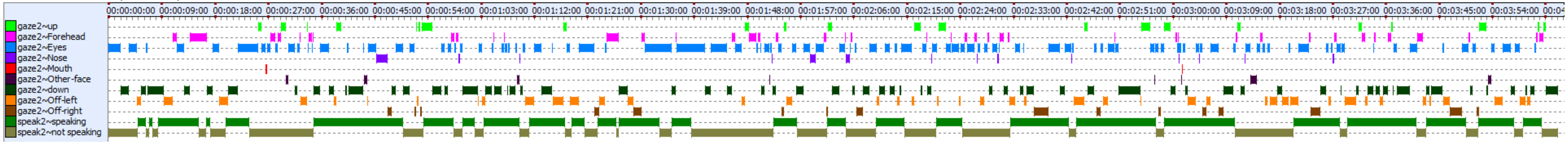

S27 with J8 (F, 17)

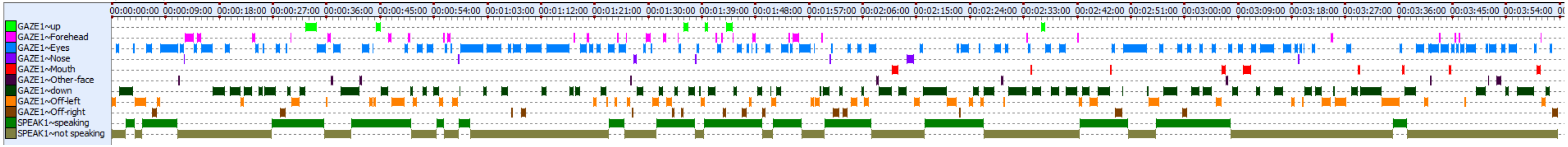

S27 with N2 (F, 35)

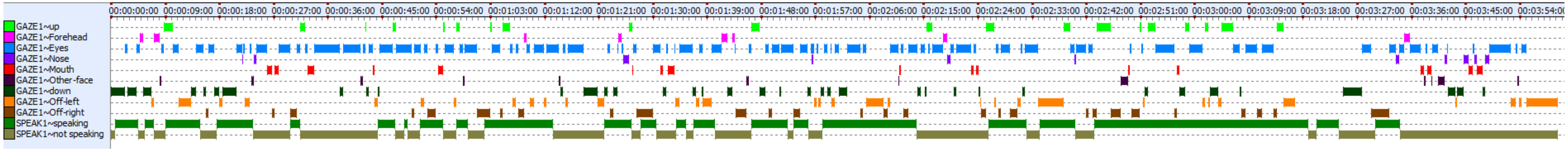

Participant I1 (M, 28)

S1 With D1 (M, 32)

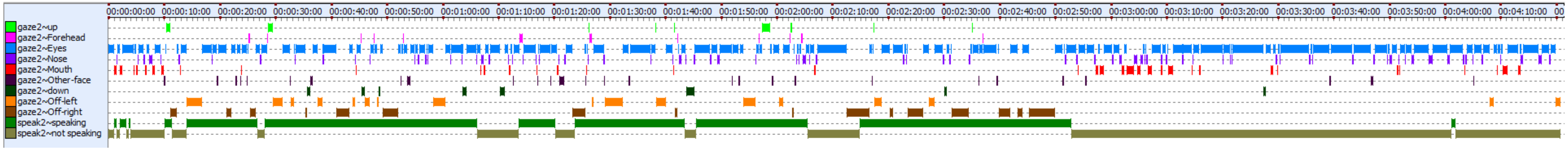

S21 with A5 (F, 18)

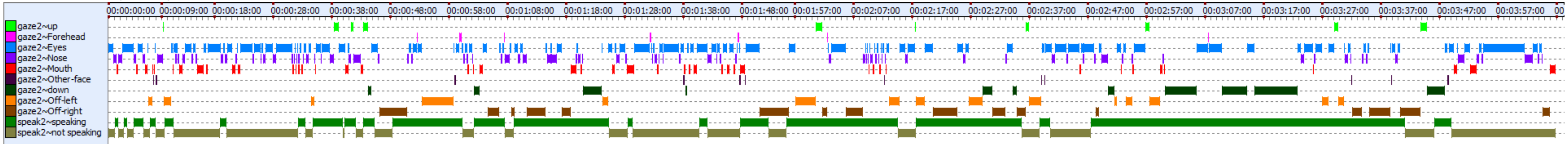

S21 with S5 (F, 39)

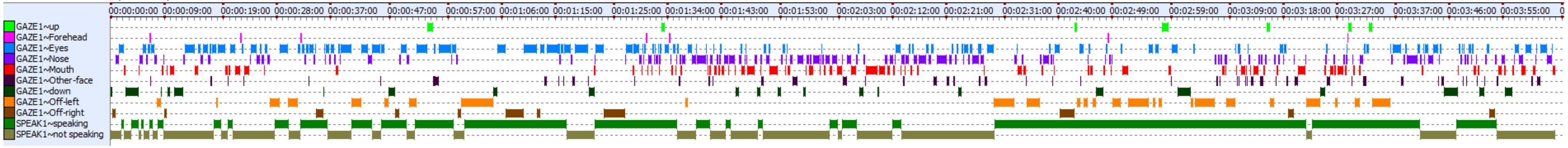

Participant I2 (M, 35)

S5 With M1 (M, 42)

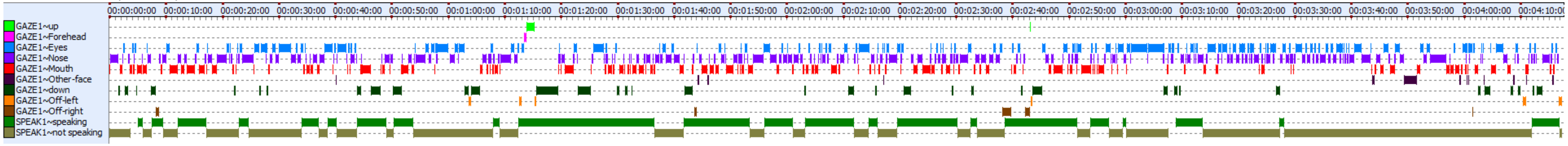

Participant J5 (F, 50)

S23 with A7 (F, 27)

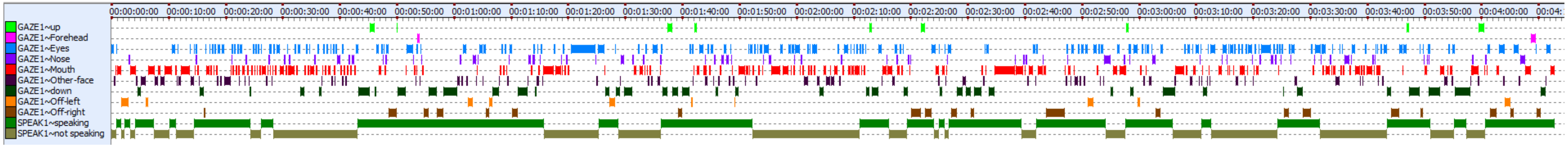

S23 with C2 (F, 20)

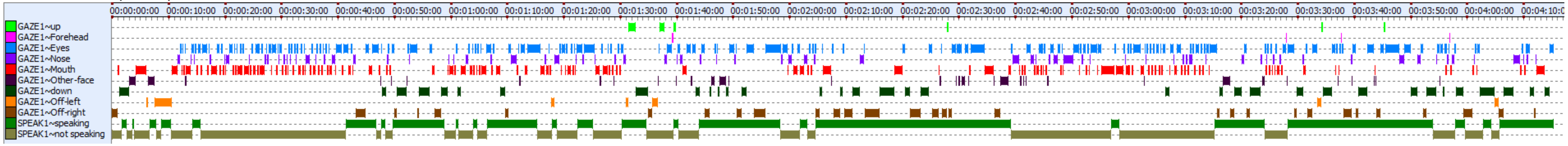

S23 with D4 (M, 45)

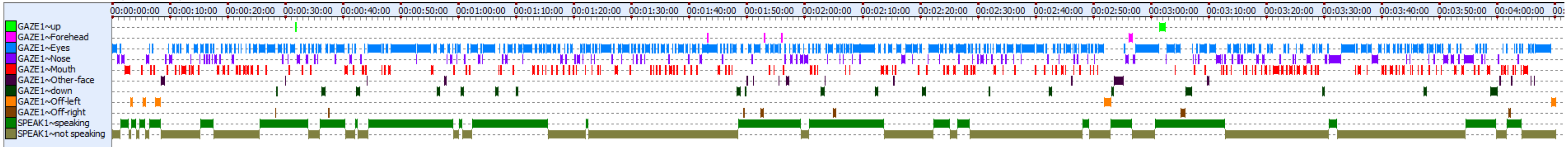

Participant J7 (F, 25)

S25 with L3 (F, 35)

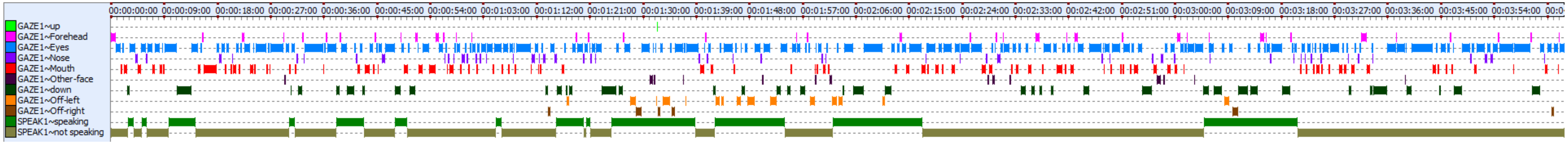

Participant J8 (F, 17)

S26 with A8 (F, 40)

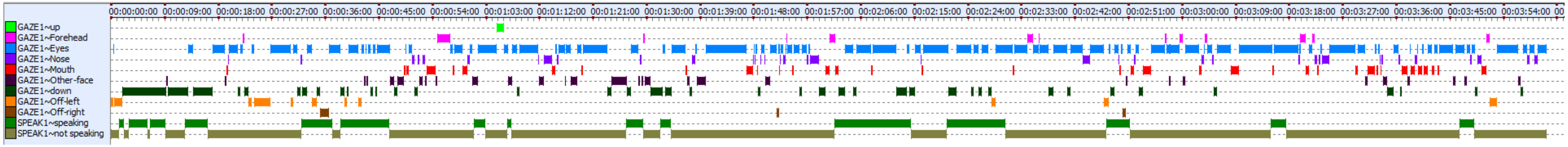

S26 with L4 (M, 25)

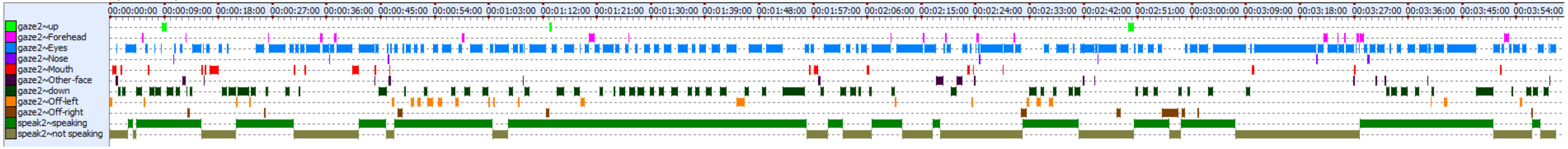

S27 with D7 (M, 19)

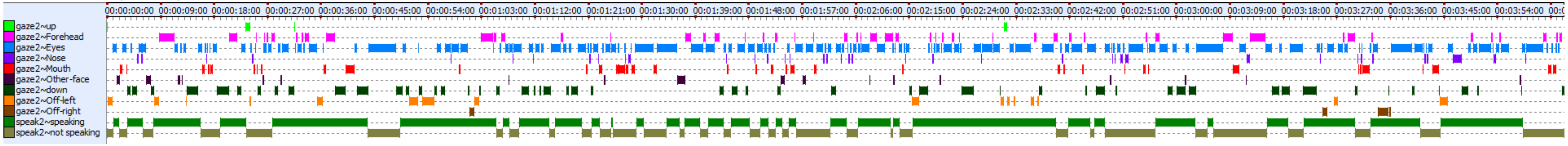

S27 with H2 (M, 20)

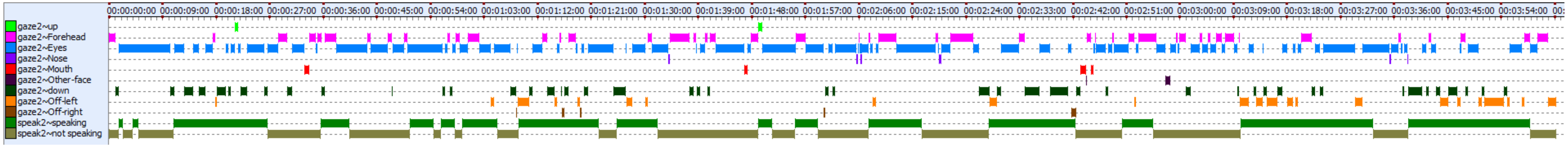

S27 with N2 (F, 35)

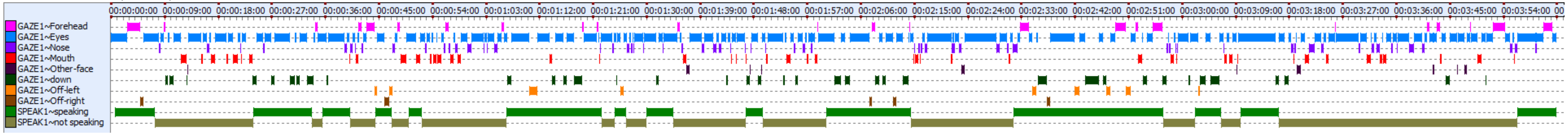

Participant L1 (M, 18)

S6 With B1 (M, 18)

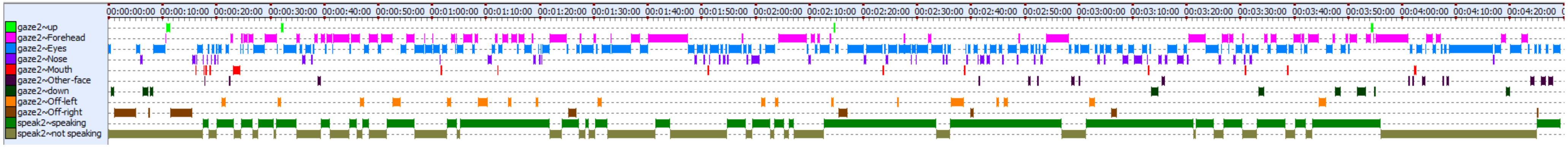

Participant L2 (F, 20)

S20 with G1 (M, 60)

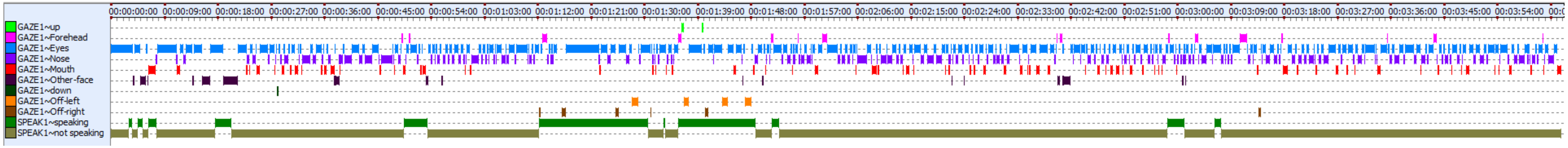

S20 with M1 (M, 42)

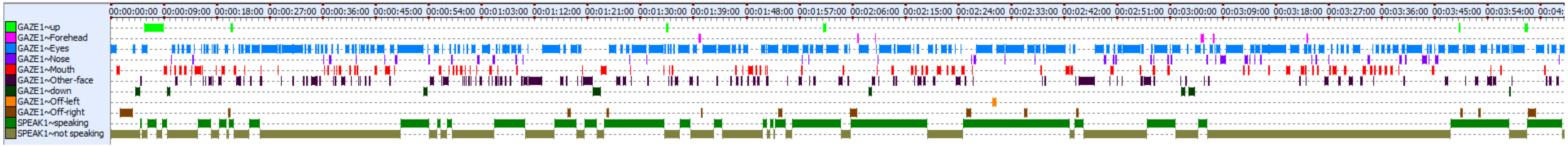

Participant L3 (F, 35)

S25 with S6 (M, 22)

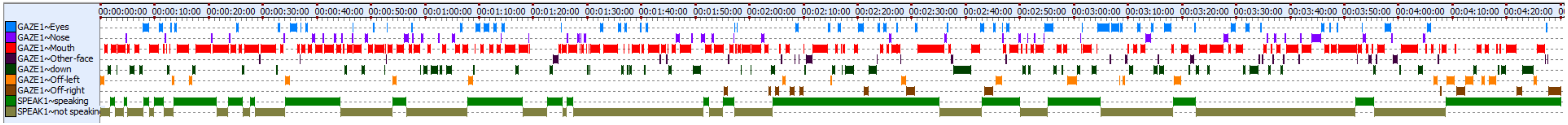

Participant L4 (M, 25)

S26 with J8 (F, 17)

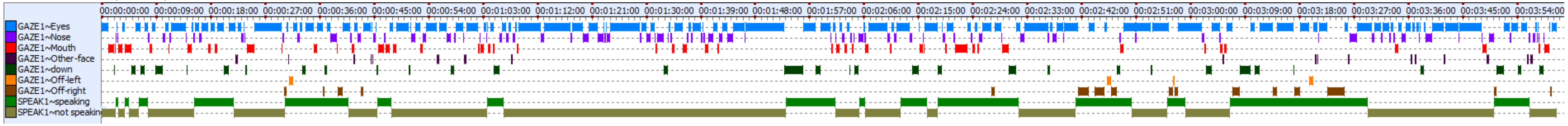

S26 with A8 (F, 40)

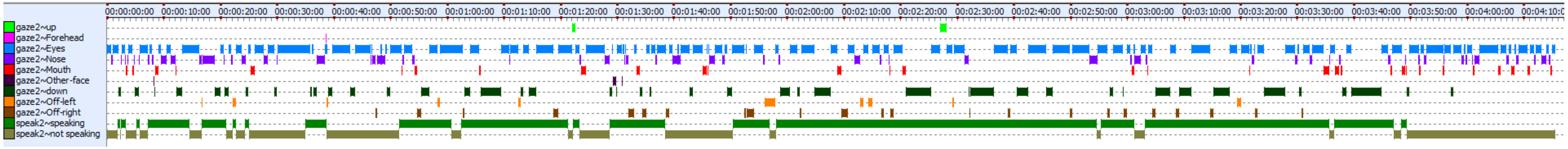

S26 with E2 (F, 40)

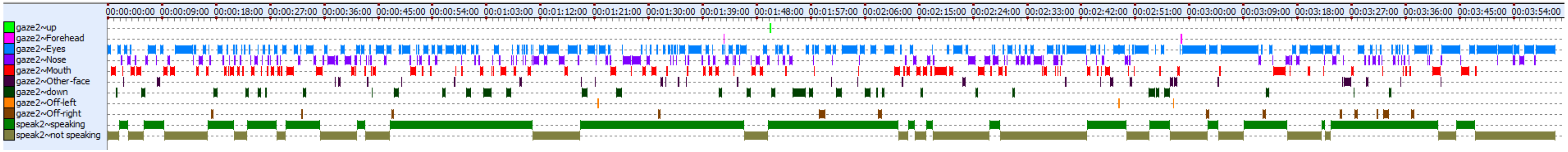

Participant L5 (M, 36)

S30 with C3 (M, 59)

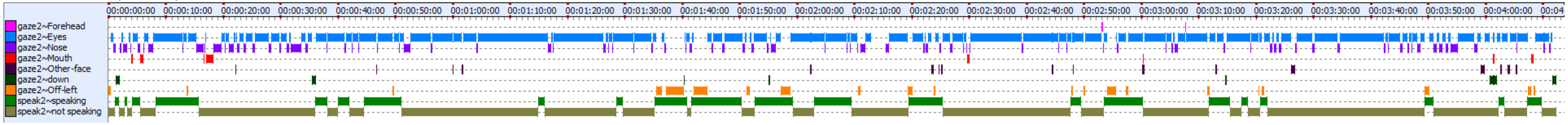

S30 with S7 (M, 48)

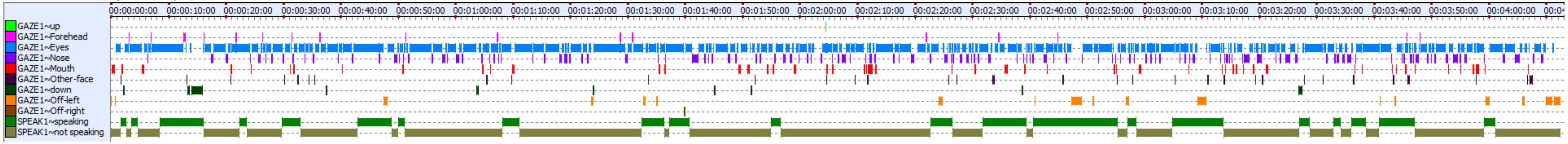

Participant M1 (M, 42)

S4 with H1 (F, 20)

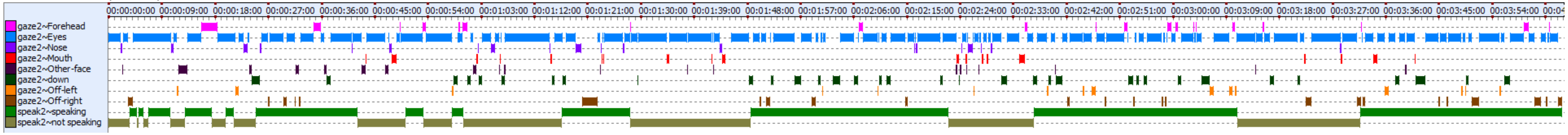

S5 With I2 (M, 35)

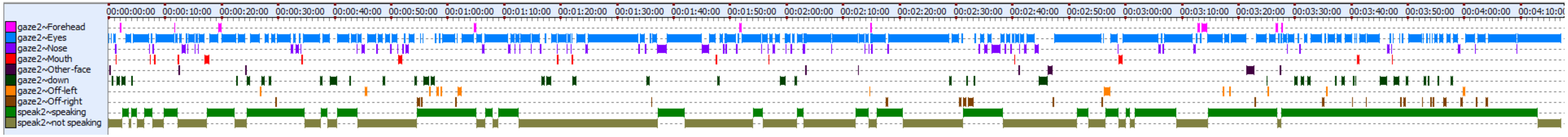

S20 with G1 (M, 60)

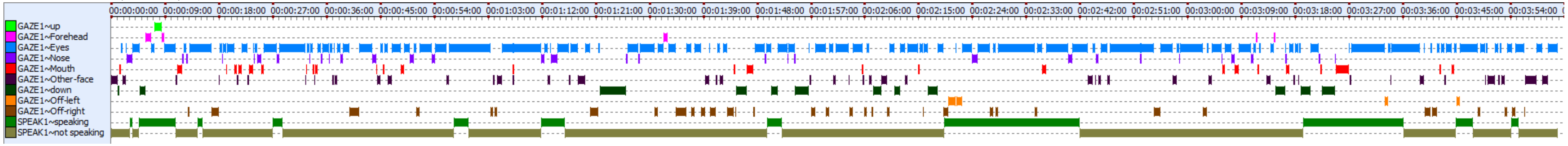

S20 with L2 (F, 20)

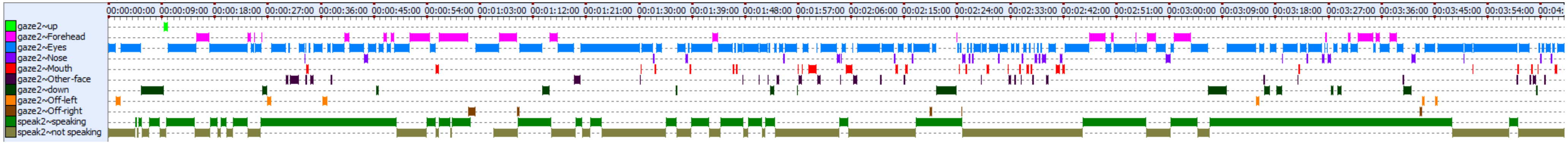

Participant M2 (M, 18)

S15 With N1 (M, 22)

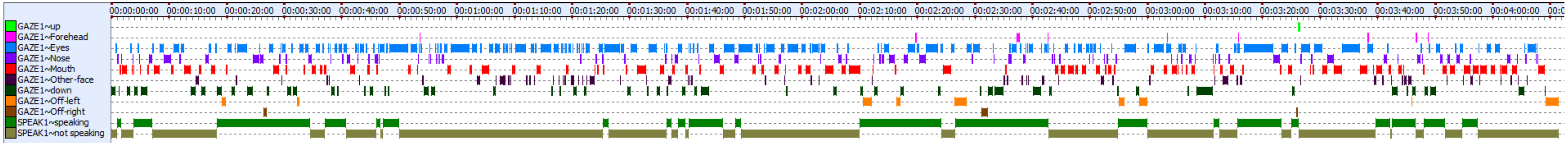

Participant N1 (M, 22)

S15 With M2 (M, 18)

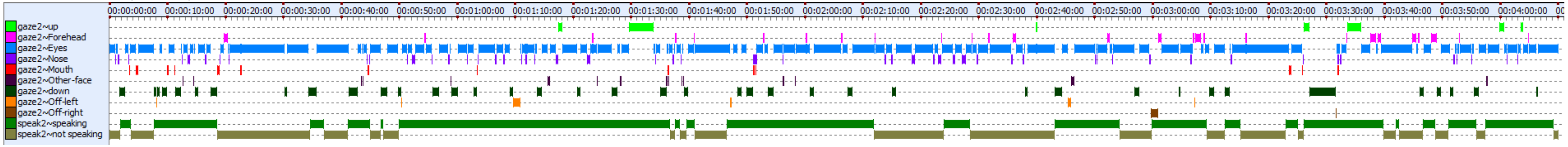

S17 With A4 (F, 50)

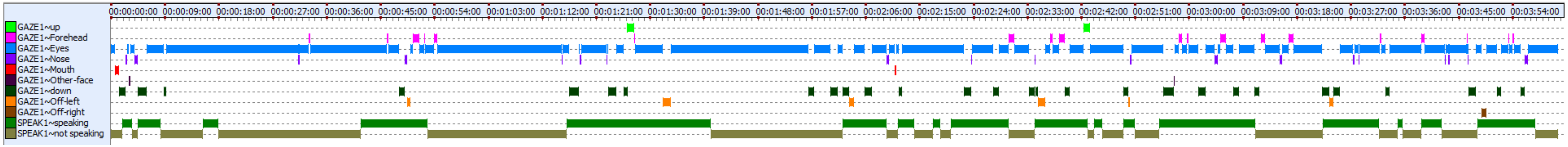

S17 With S3 (F, 22)

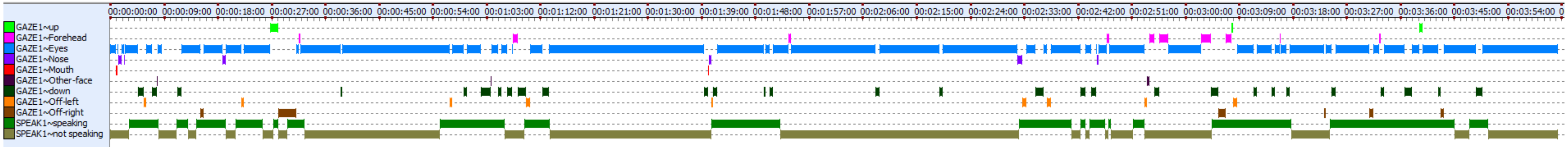

Participant N2 (F, 35)

S27 with D7 (M, 19)

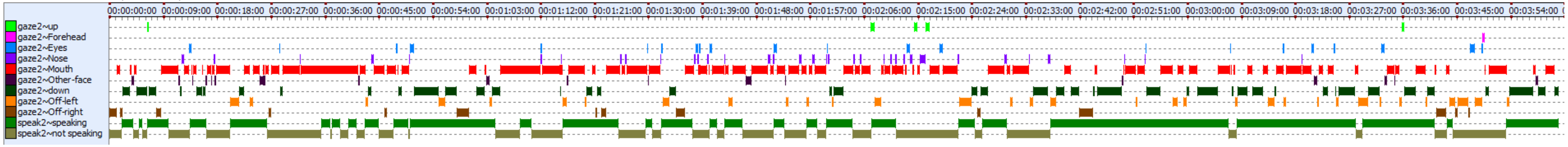

S27 with H2 (M, 20)

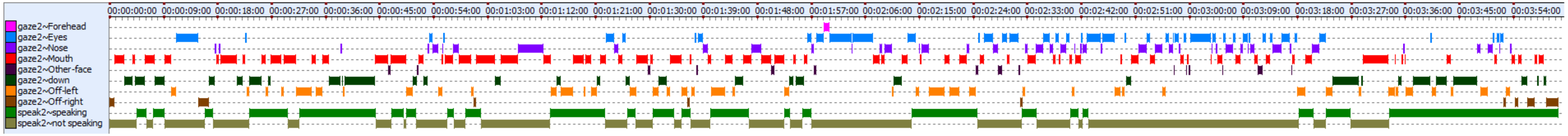

S27 with J8 (F, 17)

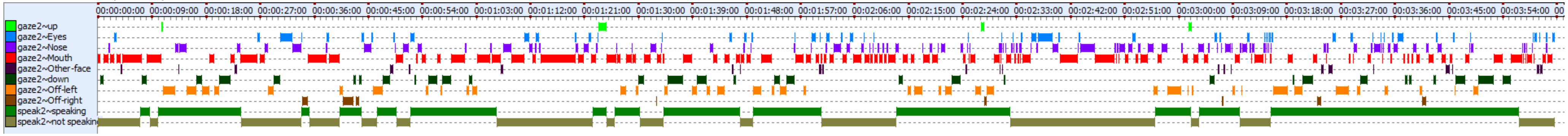

Participant P1 (F, 52)

S8 With D3 (F, 45)

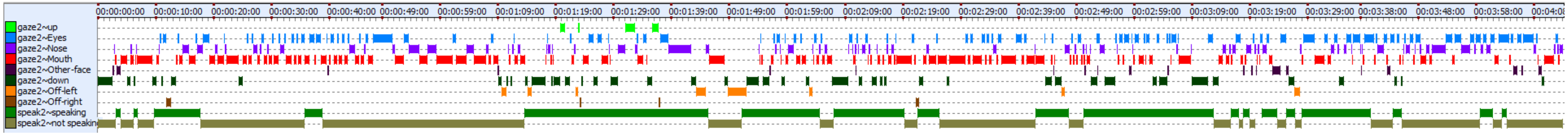

Participant P2 (M, 67)

S9 With B2 (F, 19)

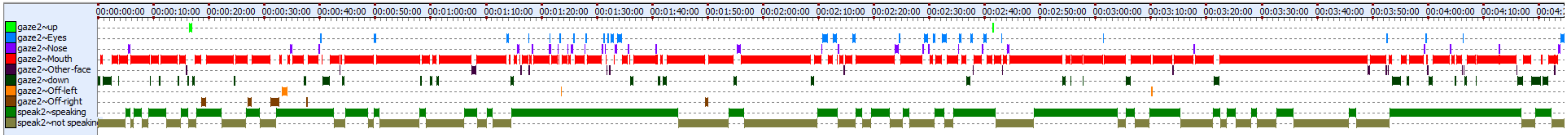

Participant R1 (F, 18)

S11 With R2 (F, 20)

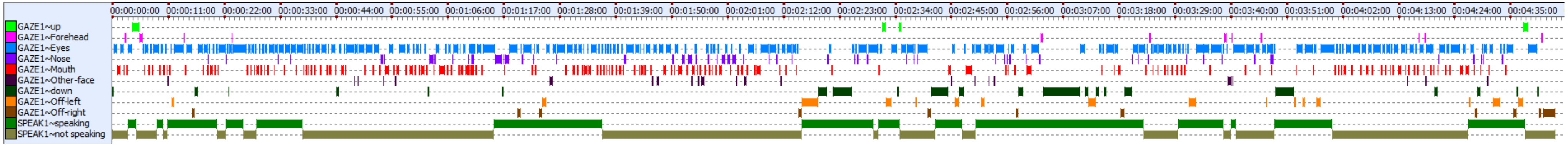

S18 with B1 (M, 18)

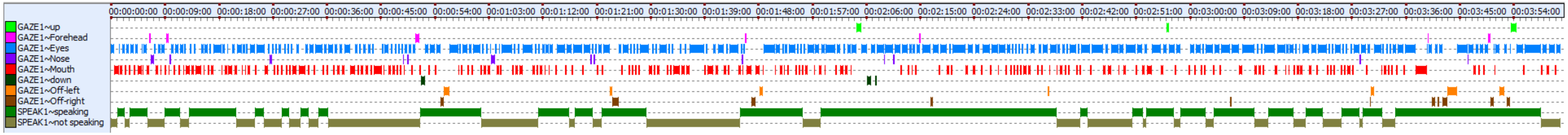

S18 with S4 (M, 29)

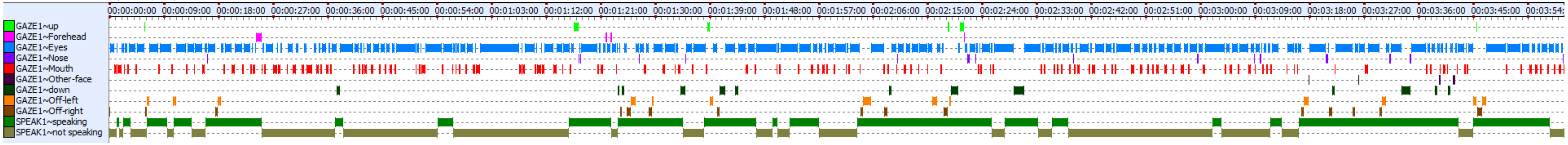

S19 with G1 (M, 60)

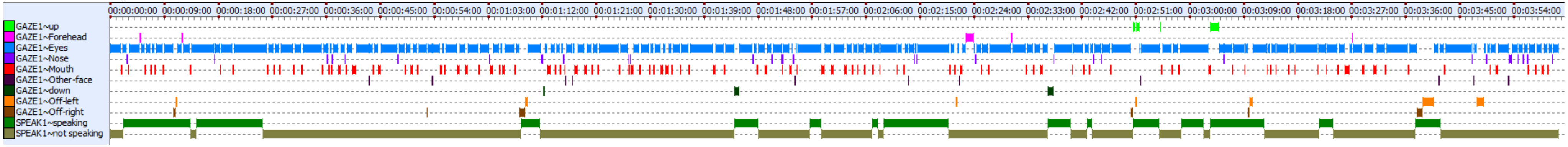

S22 with A6 (M, 23)

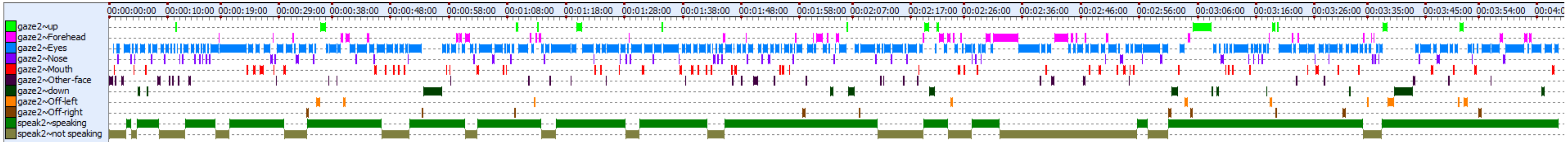

Participant R2 (F, 20)

S11 With R1 (F, 18)

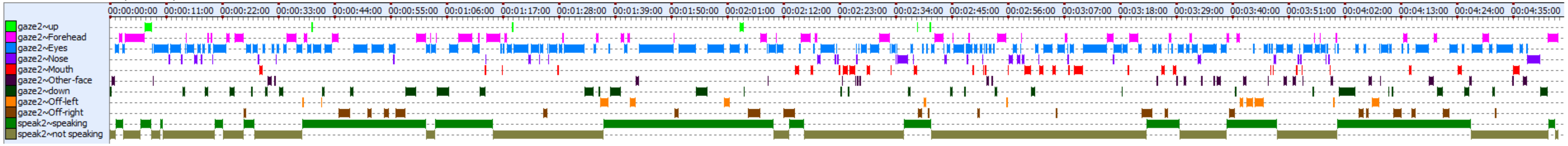

Participant R4 (M, 38)

S29 with D8 (F, 22)

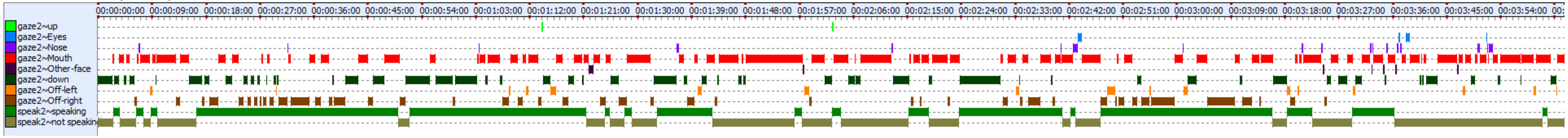

S29 with V1 (F, 45)

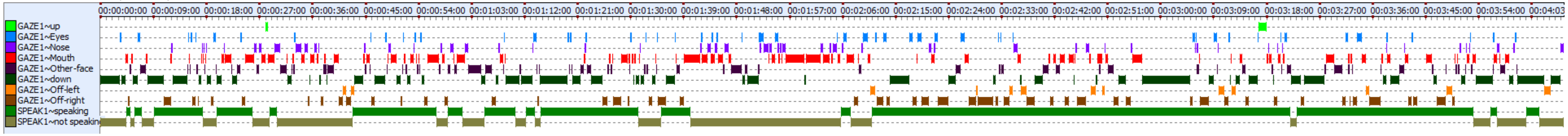

Participant S1 (F, 62)

S2 with J1 (M, 68)

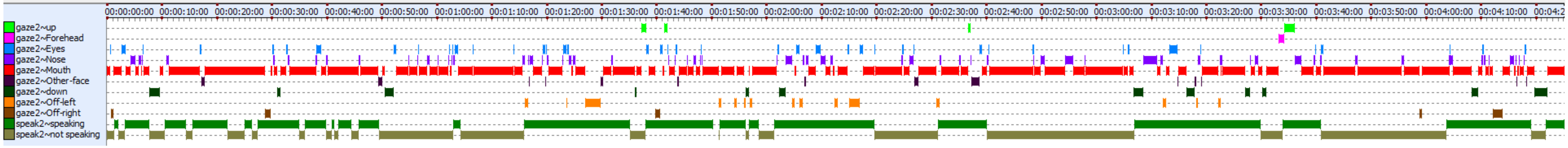

Participant S3 (F, 22)

S17 With N1 (M, 22)

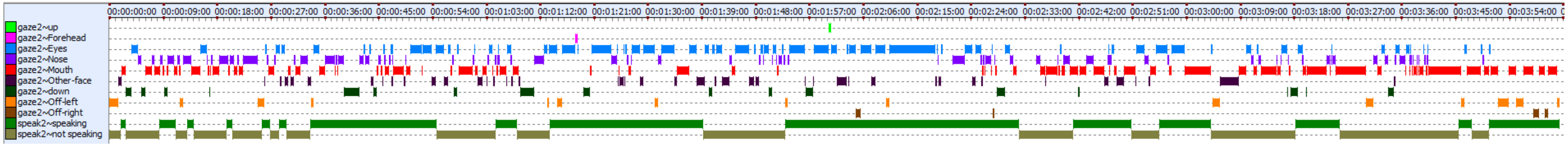

Participant S4 (M, 29)

S18 With G1 (M, 60)

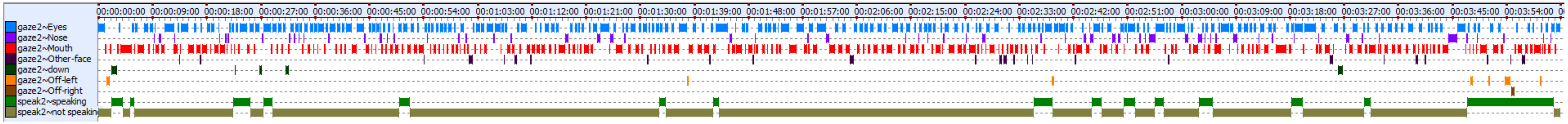

S18 with R1 (F, 18)

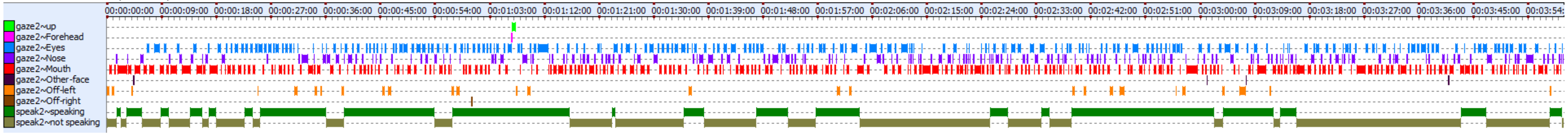

S19 with B1 (M, 18)

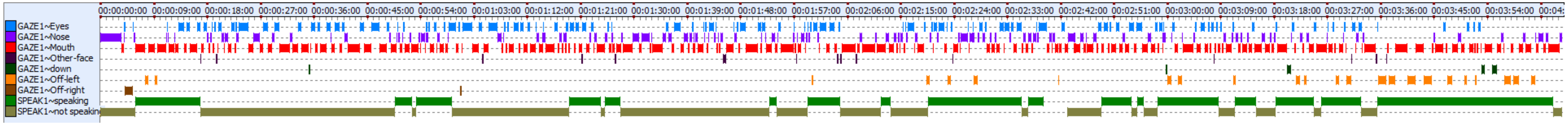

Participant S5 (F, 39)

S21 with A5 (F, 18)

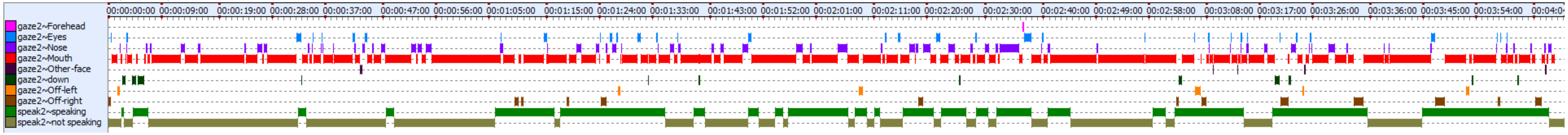

S21 with D1 (M, 32)

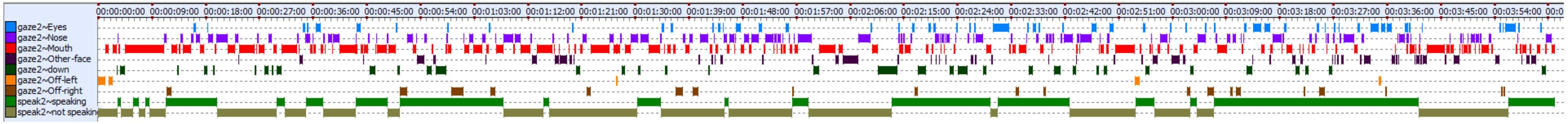

S21 with I1 (M, 28)

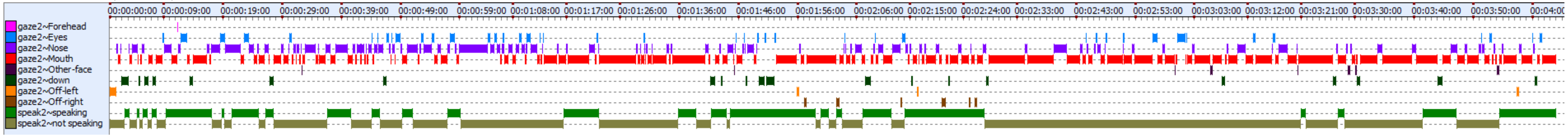

Participant S6 (M, 22)

S25 with L3 (M, 36)

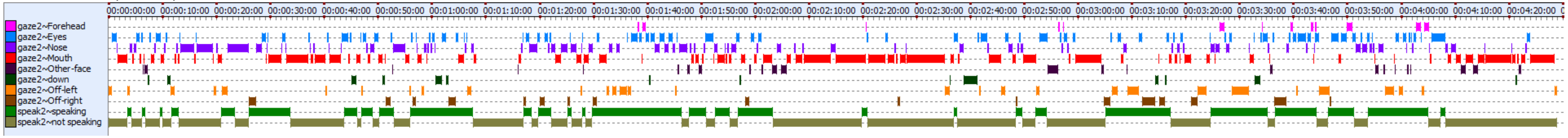

S25 with J7 (F, 25)

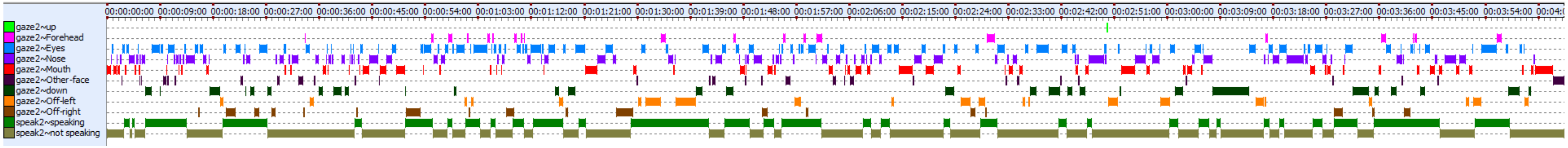

Participant S7 (M, 48)

S30 with D9 (F, 20)

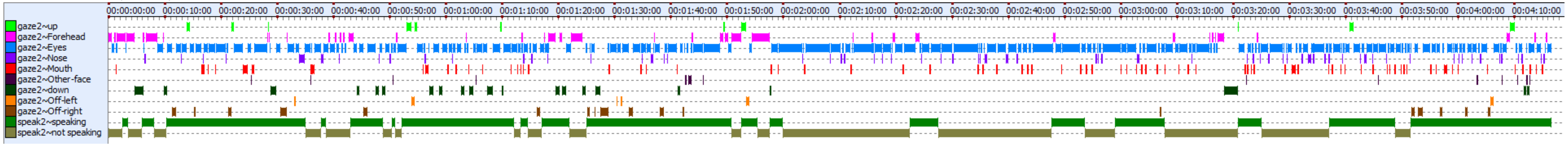

S30 with L5 (M, 36)

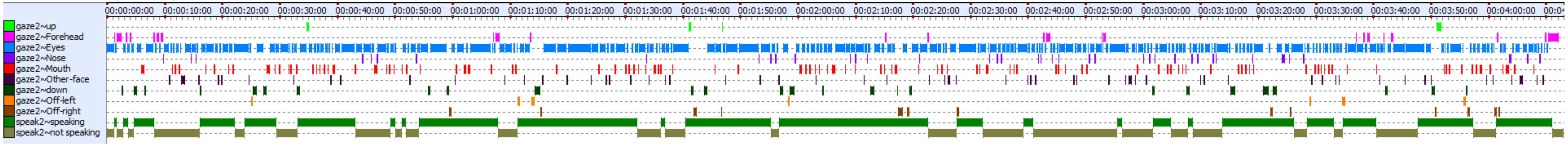

Participant V1 (F, 45)

S29 with R4 (M, 38)

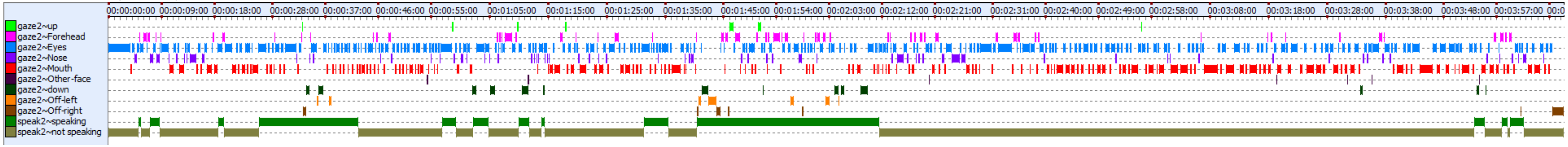

SECTION 3. Intra-class correlations

*This supplemental document for article '***Using dual eye tracking to uncover personal gaze patterns during social interaction***' provides the intra-class correlation statistics for eye gaze behaviour and speaking/listening turns. On subsequent pages below the table are graphical depictions of the ICC values that are reported in the main article results section.*

Intra-class correlations (ICCs) for a sub-set of 27 participants with gaze data for multiple conversations. Statistics are provided for assessment of consistency of the proportion of the conversation (Proportion %) spent speaking/listening or gazing on a specific location. Consistency is also assessed for the timing of speaking/listening turns and the mean duration of fixations (Timing secs) upon specific locations. Intra-class correlations are also reported for proportion of time spent looking at separate locations on the face during speaking (Proportion-Speaking %) and when listening (Proportion-Listening %). Due to unbalanced data, the ICCs were obtained using the *xtreg* command in the statistical program Stata.

Speaking and listening turns across the entire conversation

	Speaking	Listening
Proportion (%)	.30	
Timing (secs)	.38	.01

On-face and off-faze gaze across the entire conversation, and when speaking and listening

	Entire conversation		Speaking	Listening
	On-face	Off-face	On-face	Off-face
Proportion (%)	.62		.58	.57
Timing (secs)	.44	.59		

Eye fixations for separate off-face locations across the entire conversation

	up	down	left	Right
Proportion (%)	.47	.39	.64	.71
Timing (secs)	.16	.23	.30	.30

Eye fixations for separate on-face locations across the entire conversation

	eyes	mouth	nose	forehead	Other-face
Proportion (%)	.88	.80	.55	.71	.07
Timing (secs)	.73*	.69	.19	.20	.59

Eye fixations upon separate on-face locations when looking on-face, during speaking and listening

	eyes	mouth	nose	forehead	Other-face
Proportion – Speaking (%)	.88	.87	.58	.56	.15
Proportion – Listening (%)	.84	.84	.52	.74	.11

*outlier removed N1 of 4.09 secs.

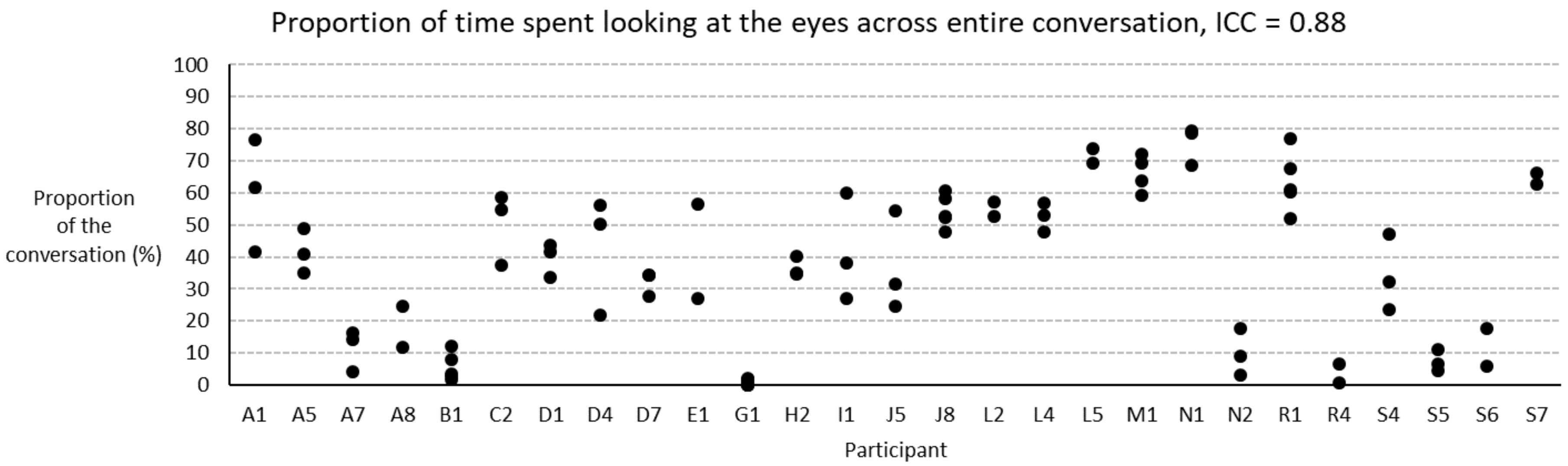
Proportion of time spent looking at the eyes across entire conversation, ICC = 0.88
Proportion of the conversation (%)
0
10
20
30
40
50
60
70
80
90
100
A1 A5 A7 A8 B1 C2 D1 D4 D7 E1 G1 H2 I1 J5 J8 L2 L4 L5 M1 N1 N2 R1 R4 S4 S5 S6 S7
Participant

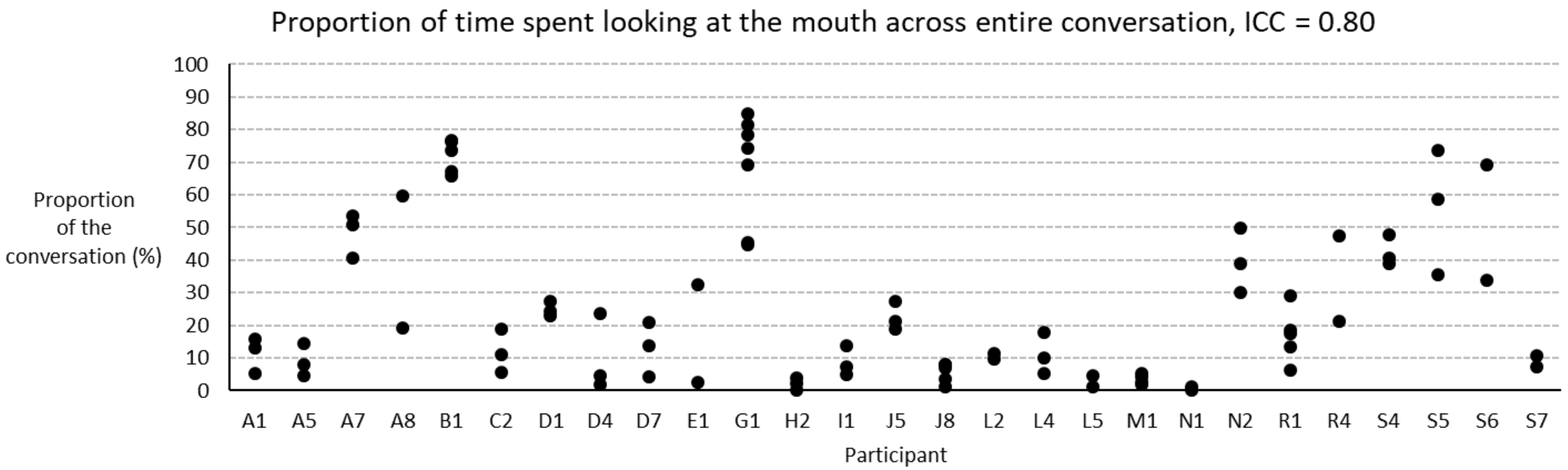
Proportion of time spent looking at the mouth across entire conversation, ICC = 0.80
Proportion of the conversation (%)
0
10
20
30
40
50
60
70
80
90
100
A1 A5 A7 A8 B1 C2 D1 D4 D7 E1 G1 H2 I1 J5 J8 L2 L4 L5 M1 N1 N2 R1 R4 S4 S5 S6 S7
Participant

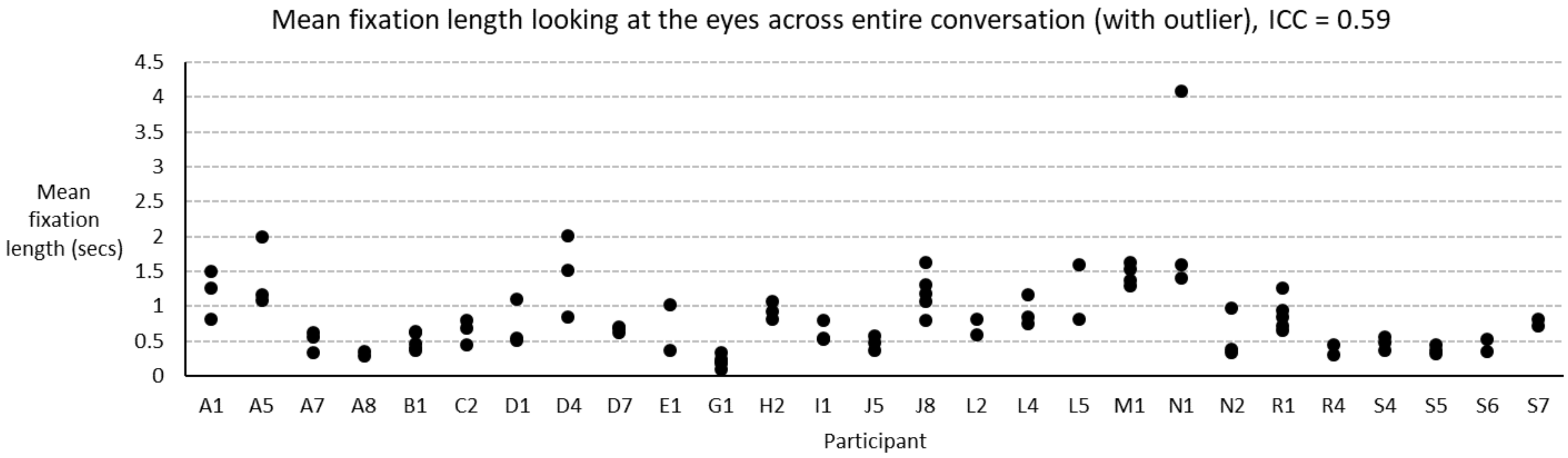
Mean fixation length looking at the eyes across entire conversation (with outlier), ICC = 0.59
Mean fixation length (secs)
4.5
4
3.5
3
2.5
2
1.5
1
0.5
0
A1 A5 A7 A8 B1 C2 D1 D4 D7 E1 G1 H2 I1 J5 J8 L2 L4 L5 M1 N1 N2 R1 R4 S4 S5 S6 S7
Participant

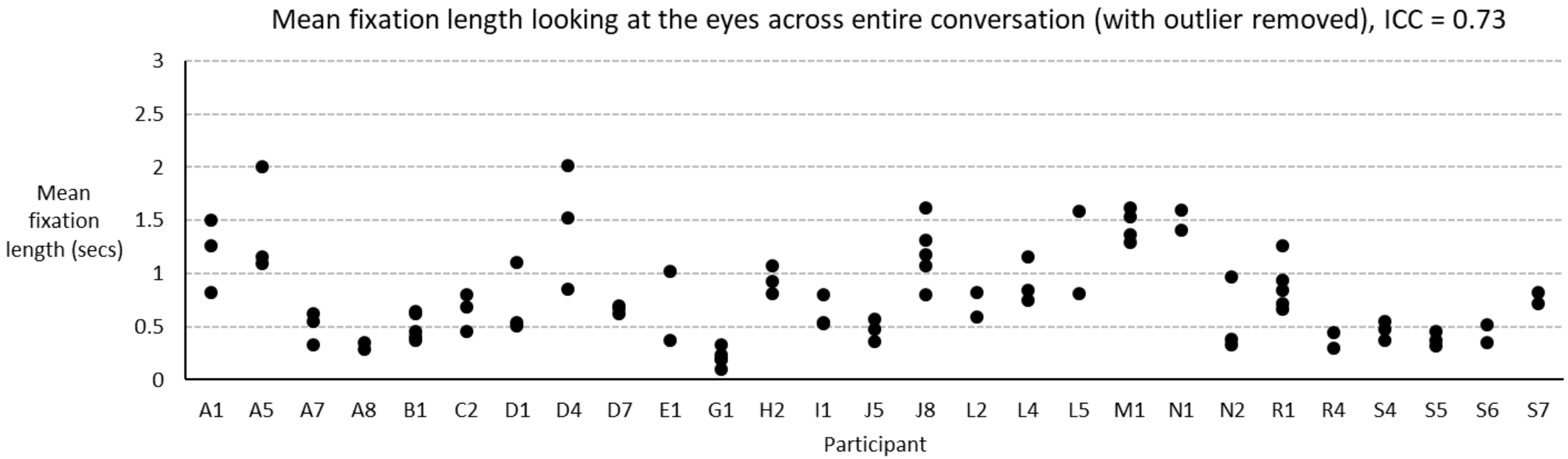
Mean fixation length looking at the eyes across entire conversation (with outlier removed), ICC = 0.73
Mean fixation length (secs)
3
2.5
2
1.5
1
0.5
0
A1 A5 A7 A8 B1 C2 D1 D4 D7 E1 G1 H2 I1 J5 J8 L2 L4 L5 M1 N1 N2 R1 R4 S4 S5 S6 S7
Participant

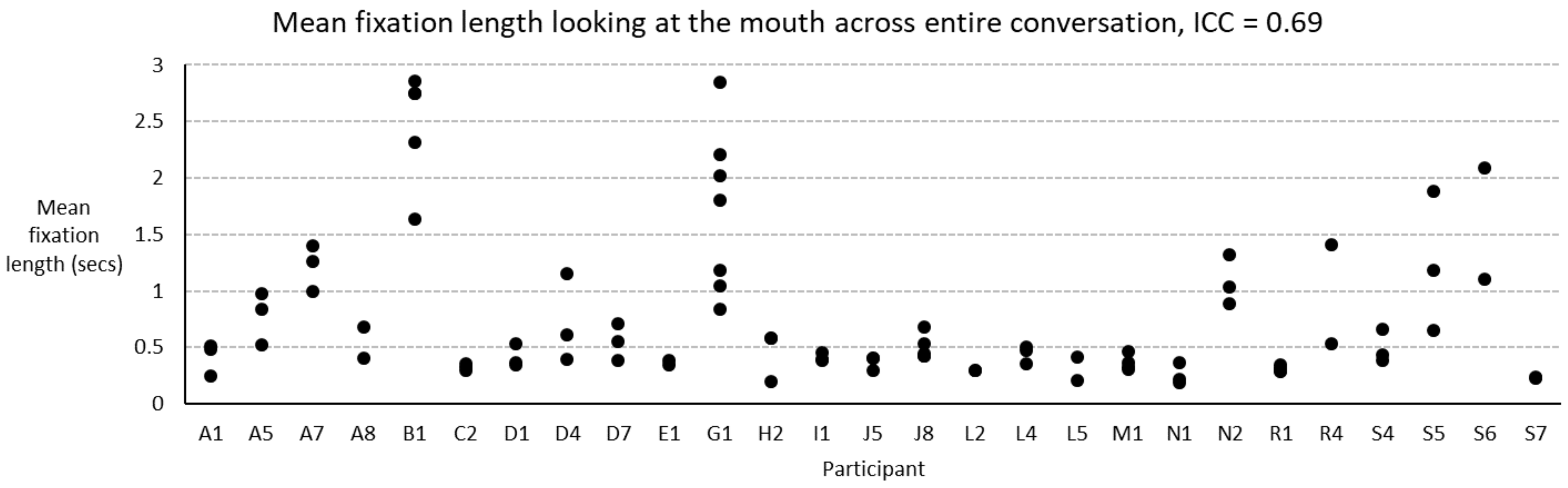
Mean fixation length looking at the mouth across entire conversation, ICC = 0.69
Mean fixation length (secs)
3
2.5
2
1.5
1
0.5
0
A1 A5 A7 A8 B1 C2 D1 D4 D7 E1 G1 H2 I1 J5 J8 L2 L4 L5 M1 N1 N2 R1 R4 S4 S5 S6 S7
Participant

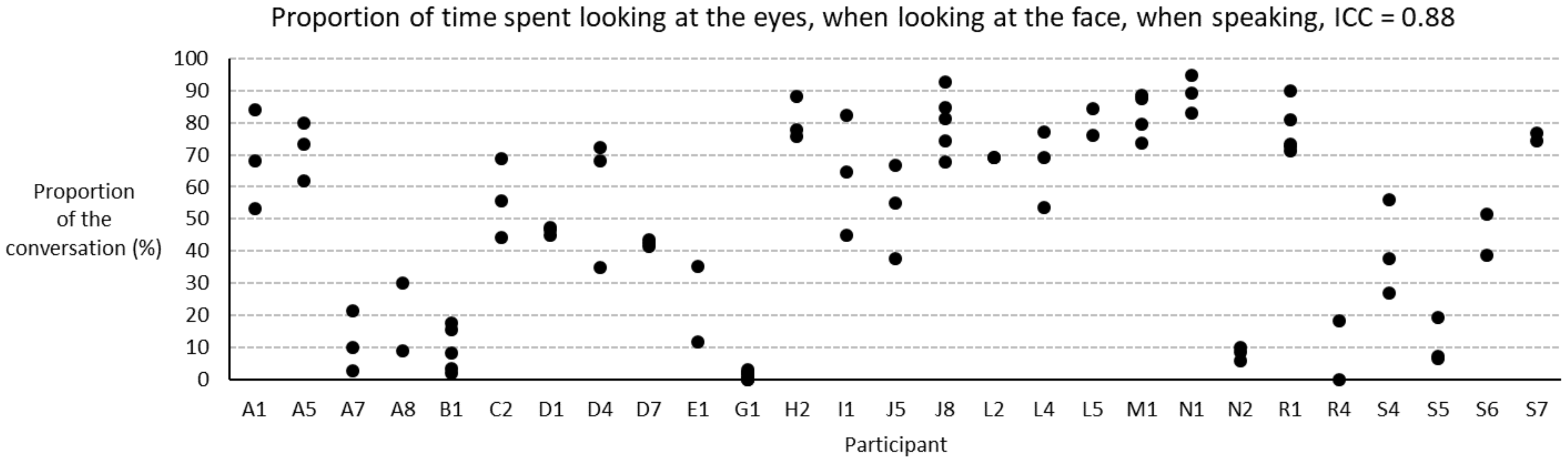
Proportion of time spent looking at the eyes, when looking at the face, when speaking, ICC = 0.88
Proportion of the conversation (%)
0
10
20
30
40
50
60
70
80
90
100
A1 A5 A7 A8 B1 C2 D1 D4 D7 E1 G1 H2 I1 J5 J8 L2 L4 L5 M1 N1 N2 R1 R4 S4 S5 S6 S7
Participant

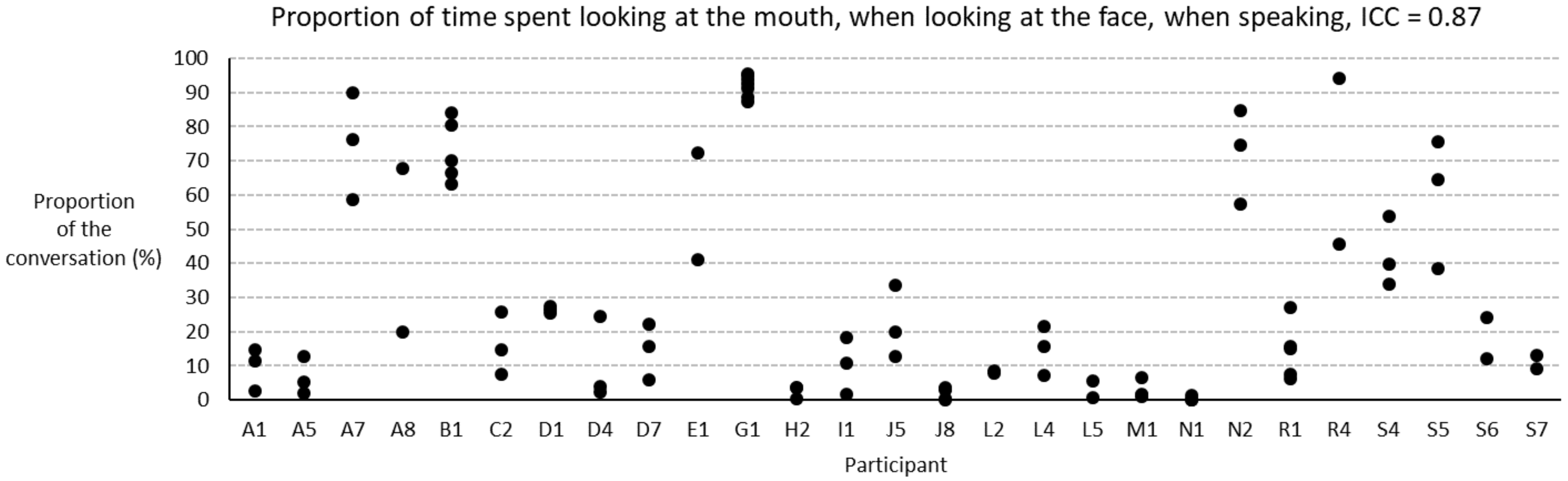
Proportion of time spent looking at the mouth, when looking at the face, when speaking, ICC = 0.87
Proportion of the conversation (%)
0
10
20
30
40
50
60
70
80
90
100
A1 A5 A7 A8 B1 C2 D1 D4 D7 E1 G1 H2 I1 J5 J8 L2 L4 L5 M1 N1 N2 R1 R4 S4 S5 S6 S7
Participant

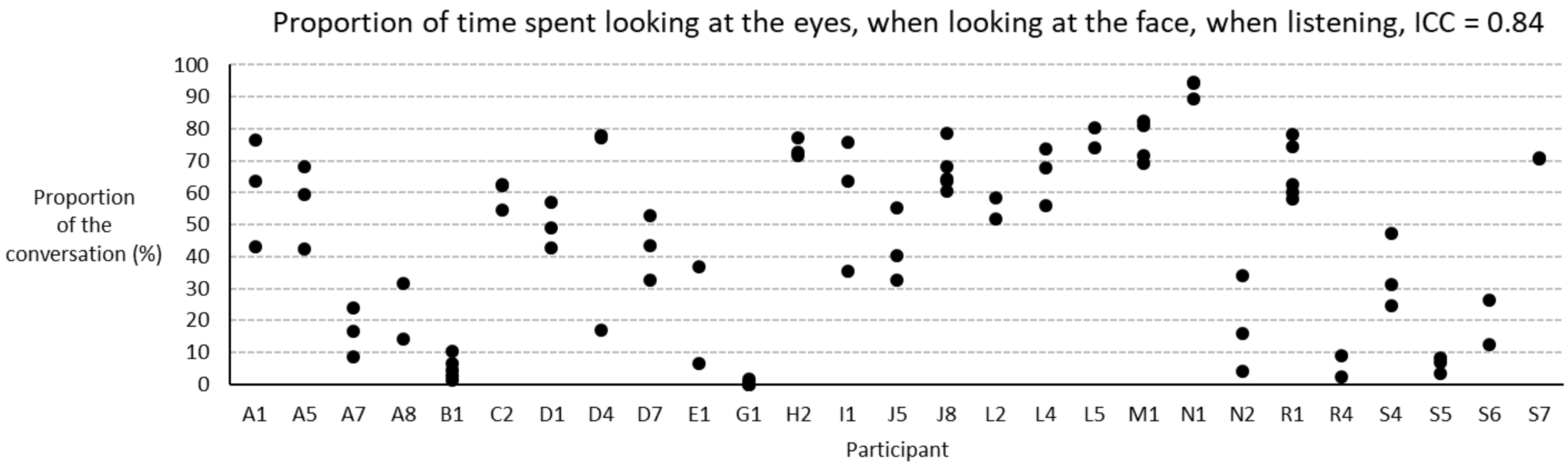

Proportion of time spent looking at the eyes, when looking at the face, when listening, ICC = 0.84
Proportion of the conversation (%)
0
10
20
30
40
50
60
70
80
90
100
A1
A5
A7
A8
B1
C2
D1
D4
D7
E1
G1
H2
I1
J5
J8
L2
L4
L5
M1
N1
N2
R1
R4
S4
S5
S6
S7
Participant

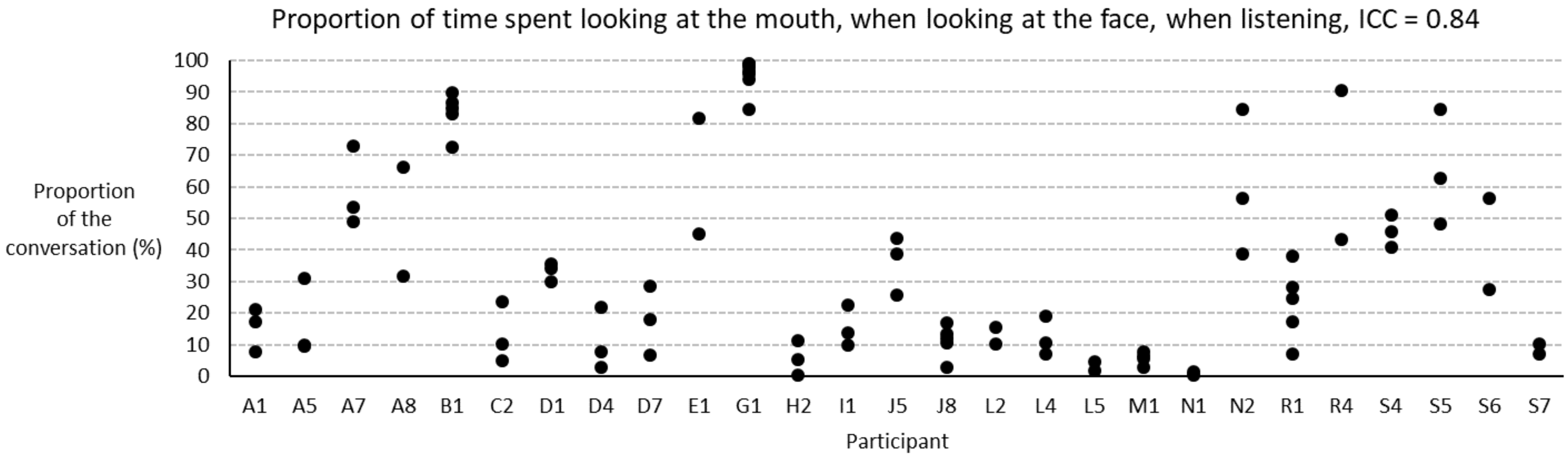

Proportion of time spent looking at the mouth, when looking at the face, when listening, ICC = 0.84
Proportion of the conversation (%)
0
10
20
30
40
50
60
70
80
90
100
A1
A5
A7
A8
B1
C2
D1
D4
D7
E1
G1
H2
I1
J5
J8
L2
L4
L5
M1
N1
N2
R1
R4
S4
S5
S6
S7
Participant

SECTION 4. Descriptive statistics

*This supplemental document for article '***Using dual eye tracking to uncover personal gaze patterns during social interaction***' provides the descriptive statistics for eye gaze behaviour and speaking/listening turns. On page 36 are scatterplots for the association between age and gaze preference that is discussed in the article.*

Descriptive statistics averaged across all 49 participants with tracking data. Provided in the table are means, with standard deviations in curved brackets, and the min-max in square brackets. Proportion (%) refers to the proportion of the conversation speaking/listening or gazing on a particular region, and Timing (secs) refers to the mean length of a typical turn/fixation.

Speaking and listening across the entire conversation

	Speaking	Listening
Proportion (%)	47 (11) [17-75]	
Timing (secs)	6.6 (2.5-16.6)	7.5 (3.7-25.8)

On-face and off-faze gaze fixation across the entire conversation, and when speaking and listening

	Entire conversation		Speaking	Listening
	On-face	Off-face	On-face	On-face
Proportion (%)	80 (10) [48-94]		71 (13) [38-90]	90 (8) [60-99]
Timing (secs)	5.4 (3.3) [1.4-21.0]	1.0 (0.3) [0.5-1.9]		

Eye fixations for separate off-face locations across the entire conversation

	up	down	left	Right
Proportion (%)	1 (2) [0-7]	10 (7) [1-29]	5 (3) [1-14]	4 (4) [1-18]
Timing (secs)	0.7 (0.3) [0.1-1.5]	0.8 (0.2) [0.4-1.3]	0.8 (0.3) [0.4-2.4]	0.7 (0.3) [0.2-1.5]

Eye fixations for separate on-face locations across the entire conversation

	eyes	mouth	nose	forehead	Other-face
Proportion (%)	36 (22) [1-81]	26 (22) [1-80]	11 (7) [1-35]	.71 [0-28]	.07 [0-11]
Timing (secs)	0.8 (0.5) [0.2-2.4]	0.7 (0.6) [0.2-0.5]	0.4 (0.1) [0.2-0.6]	0.5 (0.2) [0.1-1.1]	0.3 (0.1) [0.2-0.5]

Eye fixations upon separate on-face locations as a proportion of on-face gaze, during speaking and listening

	eyes	mouth	nose	forehead	Other-face
Proportion - Speaking	48 (27) [1-89]	29 (28) [1-92]	12 (9) [1-38]	6 (8) [0-34]	4 (3) [0-14]
Proportion - Listening	43 (26) [1-93]	34 (28) [1-95]	13 (9) [2-42]	5 (8) [0-35]	5 (4) [0-17]

The scatterplots below show the associations reported in the main article between age and proportion of on-face gaze directed towards the eyes (left plot, *Spearman r* = -35, *p* < .05) and mouth (right plot, *Spearman r* = .40, *p* < .05). Both charts reveal the associations are influenced by four older adults (participants C3, G1, P2, and S1). These participants are in the bottom right-hand corner in the left plot, and in the top right-hand corner for the right plot. When excluding these participants there is no longer any meaningful association between age and gaze preference for eyes or mouth.

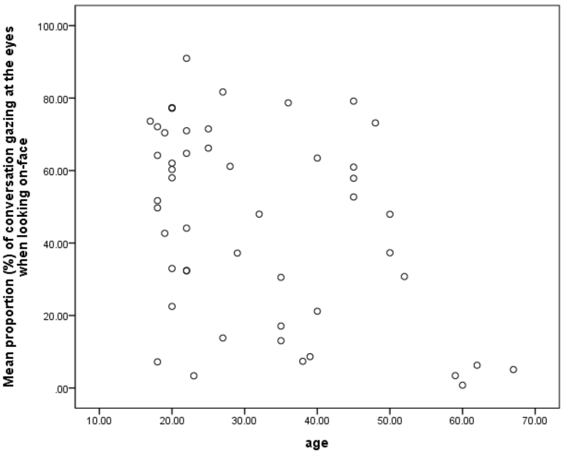

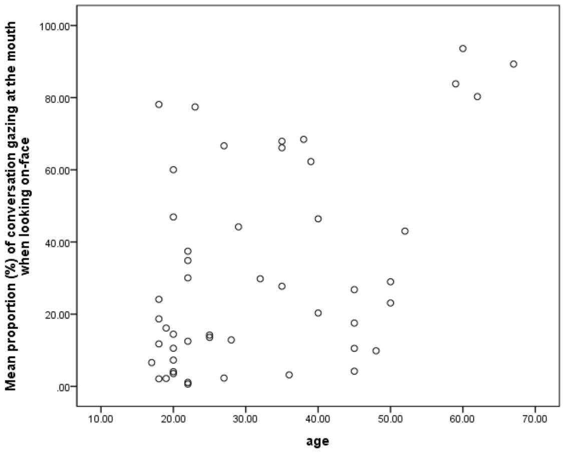

Supplement: Supplementary file 1 — Online Supplement Document [file 41598_2018_22726_MOESM1_ESM.pdf]
